# Supplementary material for: Mitogenomic evolutionary rates in bilateria are influenced by parasitic lifestyle and locomotory capacity
Source: Nat Commun. 2023 Oct 9;14:6307. doi: 10.1038/s41467-023-42095-8 (PMC10562372; doi:10.1038/s41467-023-42095-8)
Supplement: Supplementary file 1 — Supplementary Information [file 41467_2023_42095_MOESM1_ESM.pdf]

Supplementary Information

for

Mitogenomic evolutionary rates in Bilateria are influenced by parasitic lifestyle and locomotory capacity

Ivan Jakovlić, Hong Zou, Tong Ye, Hong Zhang, Xiang Liu, Chuan-Yu Xiang, Gui-Tang Wang, Dong Zhang*

*Corresponding author: Dong Zhang (dongzhang0725@gmail.com)

Supplementary Table 1. The list of mitogenomes sequenced by our team for previous studies. The "Publication" column lists the publication associated with the mitogenome.

No	Species	Phylum	Publication
1	*Pseudocapillaria tomentosa*	Nematoda	[1]
2	*Camallanus lacustris*	Nematoda	[2]
3	*Clavinema parasiluri*	Nematoda	[2]
4	*Philometra sp.*	Nematoda	[2]
5	*Camallanus cotti*	Nematoda	[3]
6	*Pingus sinensis*	Nematoda	[4]
7	*Cymothoa indica*	Arthropoda	[5]
8	*Asotana magnifica*	Arthropoda	[6]
9	*Tachaea chinensis*	Arthropoda	[7]
10	*Ichthyoxenos japonensis*	Arthropoda	[7]
11	*Tetraonchus monenteron*	Platyhelminthes	[8]
12	*Enterogyrus malmbergi*	Platyhelminthes	[9]
13	*Lamellodiscus spari*	Platyhelminthes	[10]
14	*Lepidotrema longipenis*	Platyhelminthes	[10]
15	*Sindiplozoon* sp.	Platyhelminthes	[11]
17	*Eudiplozoon* sp.	Platyhelminthes	[11]
18	*Paradiplozoon opsariichthydis*	Platyhelminthes	[11]
19	*Paratetraonchoides inermis*	Platyhelminthes	[12]
20	*Dactylogyrus lamellatus*	Platyhelminthes	[13]
21	*Thaparocleidus asoti*	Platyhelminthes	[14]
22	*Thaparocleidus varicus*	Platyhelminthes	[14]
23	*Euryhaliotrema johni*	Platyhelminthes	[15]
24	*Gangesia oligonchis*	Platyhelminthes	[16]
25	*Atractolytocestus huronensis*	Platyhelminthes	[17]
26	*Khawia sinensis*	Platyhelminthes	[17]
27	*Breviscolex orientalis*	Platyhelminthes	[17]
28	*Schyzocotyle acheilognathi*	Platyhelminthes	[17]
29	*Digramma interrupta*	Platyhelminthes	[18]
30	*Ligula intestinalis*	Platyhelminthes	[18]
31	*Gyrodactylus gurleyi*	Platyhelminthes	[19]
32	*Gyrodactylus kobayashii*	Platyhelminthes	[20]

Supplementary Table 2. Species classification and branch length in the bilaterian dataset. The dataset was classified according to two criteria: life history (LHT) and locomotory capacity (LOC). EctoP are ectoparasites, EndoP are endoparasites. The mean and standard deviation (SD) values are shown for branch lengths in each category. Source data are provided as a Source Data file.

Category	Count	Branch length	
		mean	SD
**LHT**			
**EctoP**	117	5.536	2.949
**EndoP**	276	9.052	2.654
**F**	10261	2.371	0.782

**MP**	186	2.501	0.317
**Parasitoid**	66	3.420	0.945
**LOC**			
**H**	8742	2.271	0.534
**I**	952	2.301	0.756
**L**	1212	5.050	3.018

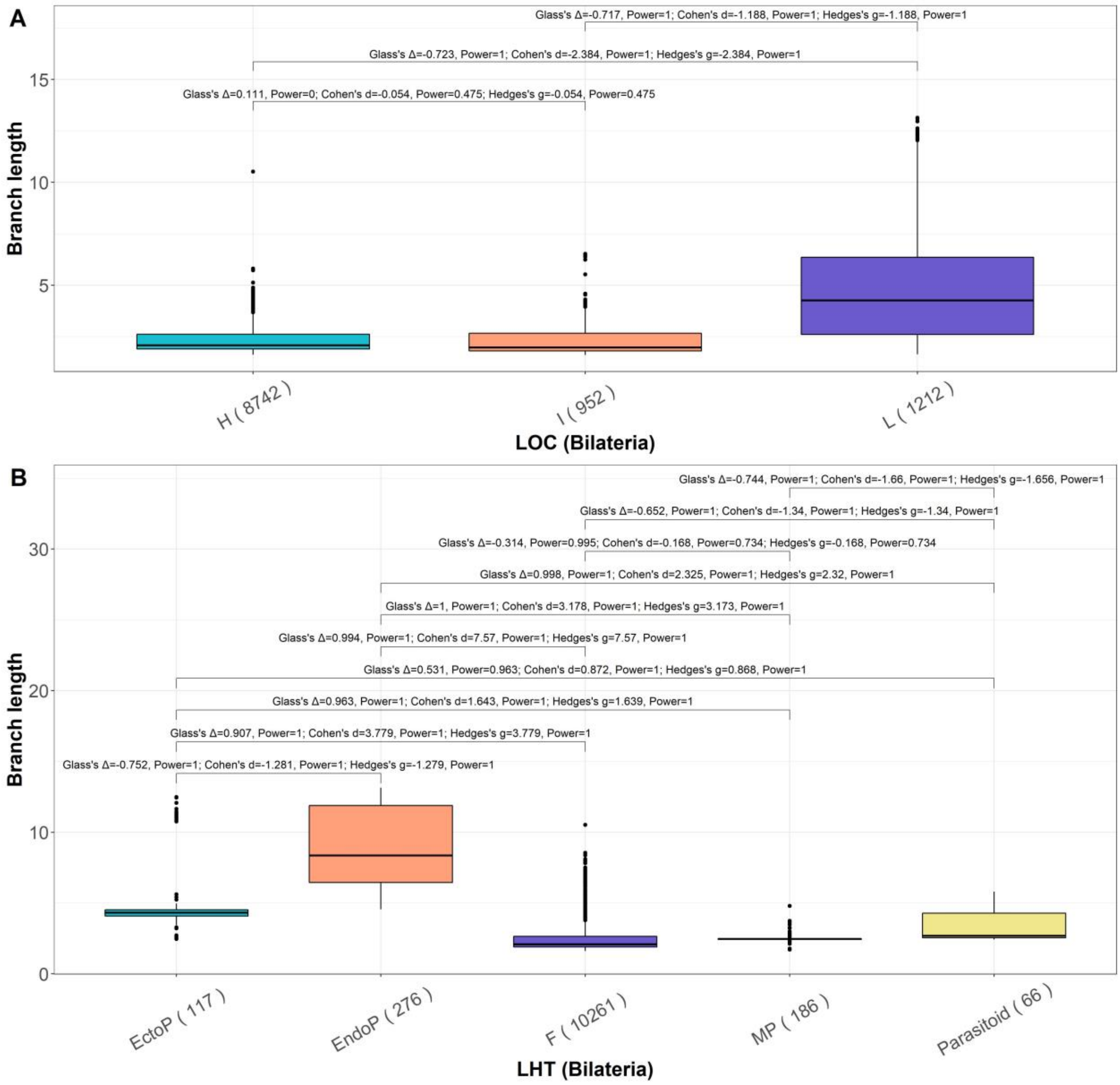

Supplementary Figure 1. The effect sizes and statistical power analyses for pairwise group comparisons. Three different effect size measures were used. Cohen's d should be used when the two groups have similar standard deviations and the same sample size that is >20. Glass's Δ should be used when the two groups' standard deviations are different. Hedges's g should be used when the two groups have similar standard deviations and different sample sizes, or both groups have a

sample size <20 per group. Cohen's d values can be interpreted in the following way: $d<0.2$ = small effect size, $0.2<d<0.5$ = medium, $0.5<d<0.8$ = large, and $d>0.8$ = very large. In the life-history categorisation (LHT), F is free-living, EndoP is endoparasites, EctoP is ectoparasites, and MP is micropredators. In the locomotory capacity categorisation (LOC), H is High, I is Intermediate, and L is Low. Statistical power analyses were conducted for each effect size measure using pwr.t2n.test method in the R package 'pwr'. Power refers to the probability of detecting a statistically significant effect if one exists, where the commonly accepted cut-off value for a good statistical power is ≥ 0.8. Source data are provided as a Source Data file.

Supplementary Table 3. Branch lengths, and categorisation of life history and locomotory for each phylum represented in the dataset. brl is the average branch length for a phylum. IO columns show independent origins of endoparasitism (EndoP) and ectoparasitism (EctoP). Other columns show ratios of categories in a phylum, where EndoP are endoparasites, EndoP+EctoP are endoparasites+ectoparasites, MP are micropredators, and L and H LOC is low and high locomotory capacity category respectively. Source data are provided as a Source Data file.

Phylum	brl	EndoP IO	EctoP IO	EndoP	EndoP+EctoP	Parasitoid	MP	L LOC	H LOC
Acanthocephala	8.009	1		1	1	0	0	1	0
Annelida	2.989			0	0	0	0.060	0.300	0
Arthropoda	2.898	2	12	0.003	0.041	0.022	0.042	0.078	0.866
Brachiopoda	4.447			0	0	0	0	1	0
Bryozoa	3.747			0	0	0	0	1	0
Chaetognatha	3.554			0	0	0	0	0	0
Chordata	1.965			0	0	0	0.002	0.009	0.892
Echinodermata	2.035			0	0	0	0	0.991	0
Entoprocta	2.422			0	0	0	0	1	0
Gastrotricha	3.693			0	0	0	0	1	0
Gnathostomulida	5.560			0	0	0	0	1	0
Hemichordata	1.725			0	0	0	0	0.835	0
Kinorhyncha	5.525			0	0	0	0	1	0
Mollusca	3.590			0	0	0	0	0.845	0.067
Nematoda	6.838	5-7		0.804	0.804	0	0	1	0
Nematomorpha	5.530			0	0	1	0	0	0
Nemertea	2.654			0	0	0	0	1	0
Onychophora	2.343			0	0	0	0	0	0
Orthonectida	9.434	1		1	1	0	0	1	0
Phoronida	2.485			0	0	0	0	1	0
Platyhelminthes	11.397	2-3	1	0.812	0.964	0	0	1	0
Priapulida	2.303			0	0	0	0	1	0
Rotifera	5.844			0	0	0	0	1	0

Tardigrada	3.686	0	0	0	0	1	0
Xenacoelomorpha	2.936	0	0	0	0	1	0

Supplementary Note 1. The average branch length.

As a result of relatively short-branched Chordata and Arthropoda comprising most of the dataset, the average branch length calculated across all lineages was also very low (2.58), so most phyla exhibited significantly elevated evolutionary rates. The average branch length across the averages of all phyla was 4.3.

Due to this uneven sampling of species, results presented in Supplementary Table 4 may appear counterintuitive compared to the average branch lengths presented in Figure 1 in the main manuscript and Supplementary Table 3, but readers should keep in mind that the phylum tested was excluded from the rest of the dataset for each of these comparisons (i.e. each analysis was conducted on a different dataset, because a different phylum was excluded each time).

Supplementary Table 4. Statistical comparisons (two-sided t-test) of branch lengths in individual phyla vs. all other lineages in the dataset. Only phyla with 5 or more species are included. Source data are provided as a Source Data file.

Phylum	species number	t statistic	p value
Chordata	6228	-59.8282	0
Nematoda	174	42.6807	0
Platyhelminthes	151	111.2834	0
Mollusca	555	17.2342	1.09E-65
Arthropoda	3504	16.0259	3.78E-57
Acanthocephala	13	13.7862	7.06E-43
Echinodermata	107	-3.9767	7.03E-05
Brachiopoda	5	2.9129	0.0036
Annelida	100	2.8540	0.0043
Bryozoa	8	2.3013	0.0214
Chaetognatha	5	1.5166	0.1294
Hemichordata	6	-1.4665	0.1426
Xenacoelomorpha	10	0.7806	0.4351
Nemertea	19	0.2176	0.8278

Supplementary Note 2. Independent origins of parasitism

Independent origins of parasitism were inferred by inferring the ancestral states using the Maximum Likelihood method in BayesTraits v4.0.1 [21], and then manually inspecting the phylogenetic tree and identifying sister groups to parasitic lineages. This method is prone to

phylogenetic artefacts and incomplete sampling-caused errors, so we also relied on published literature to confirm our trait state mapping. In some cases, the evolutionary scenarios were rather straightforward (e.g. a single origin in Acanthocephala), but in some cases, the evolutionary history of parasitism is very complex, so the ancestral mode is difficult to infer. Nematoda are an example. For this lineage, we inferred 7 independent origins of endoparasitism following the scenarios of the evolution of parasitism proposed for this lineage in previous studies [22–24]: in Trichinellida, Mermithida, Spirurina, Steinernematidae, Heterorhabditidae, and two in Strongylida. Another example is Platyhelminthes, where there can be 2 or 3 origins of endoparasitism, depending on the unresolved relationships between Monogenea, Cestoda and Trematoda. In Arthropoda, we identified two origins of endoparasitism (Rhinonyssidae and Pentasomida) and 12 independent origins of ectoparasitism: Lernaeidae, Psoroptidae, Ergasilidae, Caligidae, Bopyridae, Cymothoidae+Corallanidae, Hippoboscidae+Streblidae, Siphonaptera, Phthiraptera, Argulidae, Varroidae, and Ixodida.

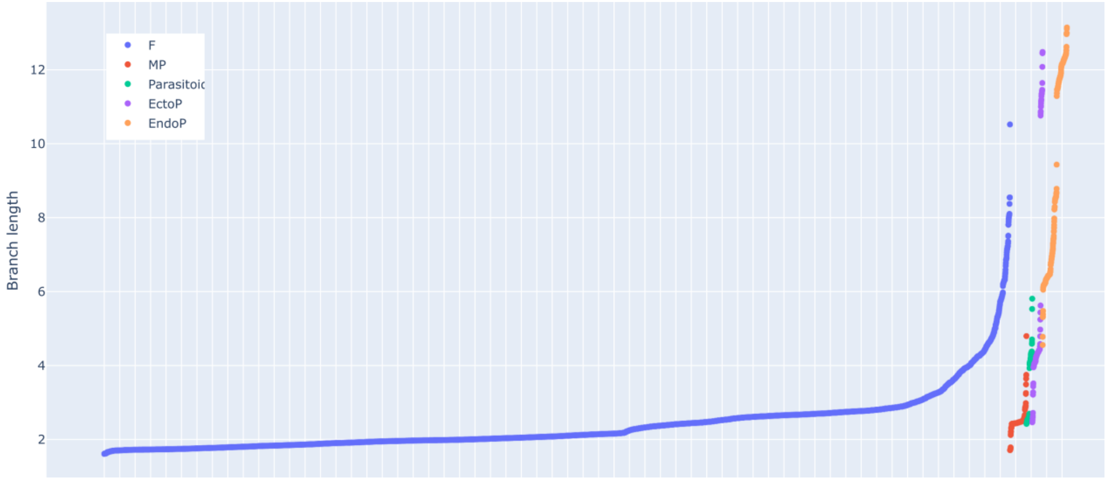

Supplementary Figure 2. Distribution of branch lengths across different life history categories. Source data are provided as a Source Data file.

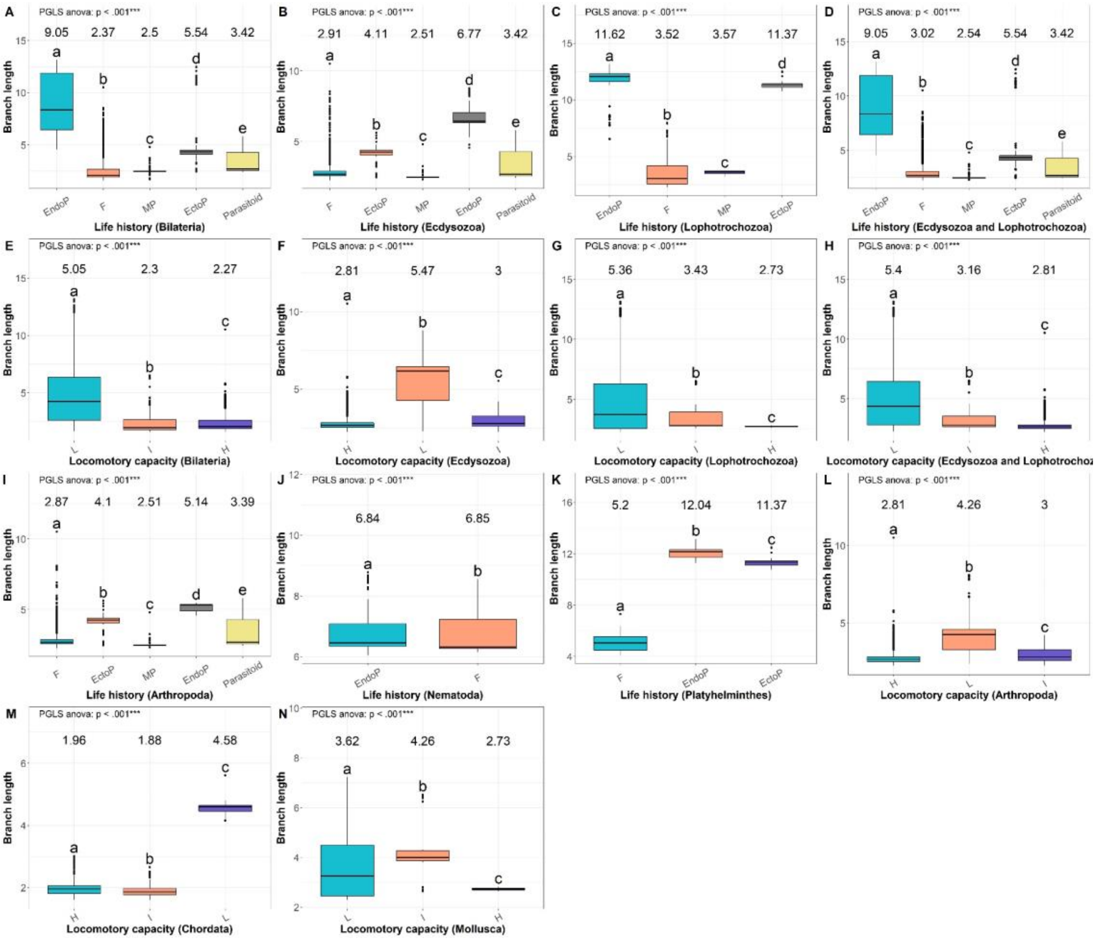

Supplementary Figure 3. PGLS ANOVA pairwise comparisons between groups and overall comparisons. Average branch length values are shown above the boxplots. Different letters above the box plots indicate statistically significant differences. In the life-history categorisation, F is free-living, EndoP is endoparasites, EctoP is ectoparasites, and MP is micropredators. In the locomotory capacity categorisation, H is High, I is Intermediate, and L is Low. Overall PGLS ANOVA results are shown in the upper left corner. Different letters above the boxplots indicate statistically significant differences (p<0.05). Source data are provided as a Source Data file.

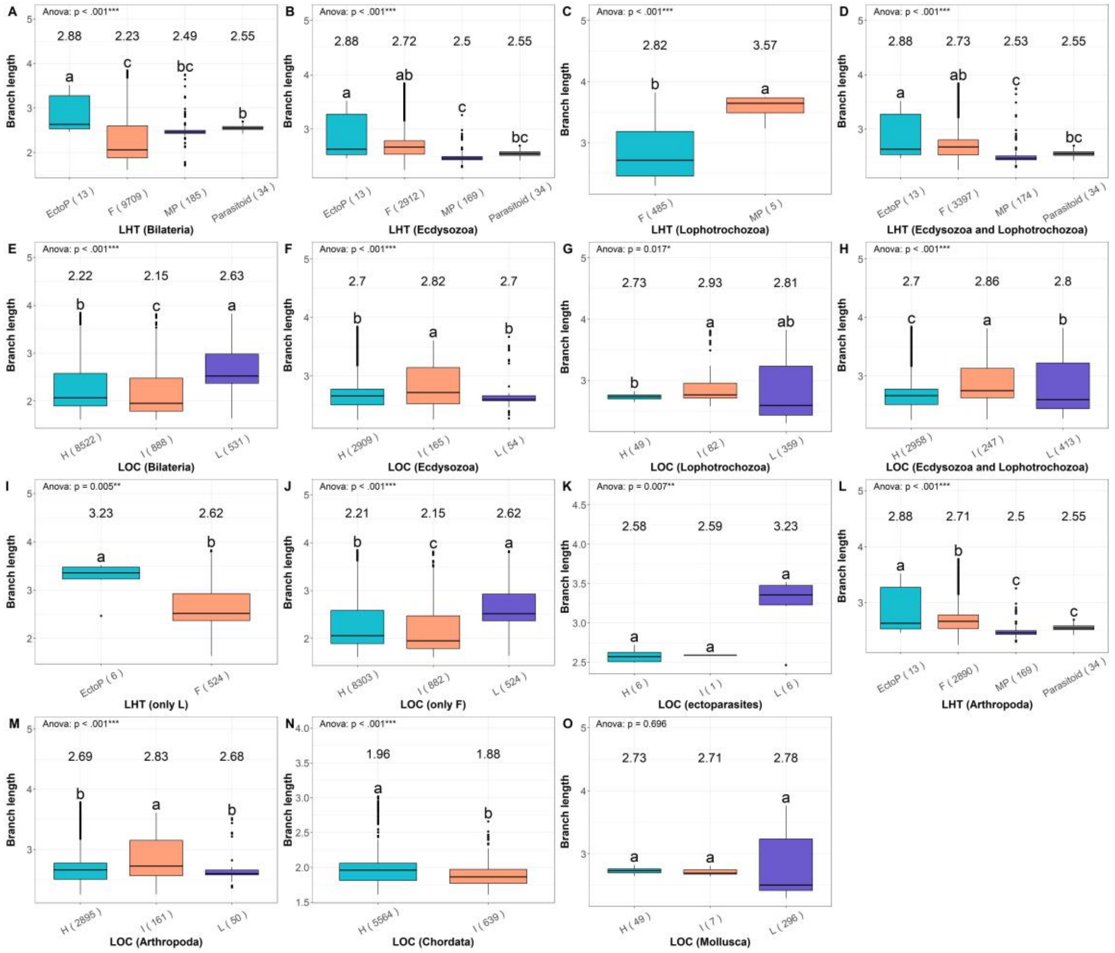

Supplementary Figure 4. Comparison of average branch lengths in different life history (LHT) and locomotory capacity (LOC) categories at the Bilateria level with outliers removed. See Supplementary Figure 3 for other details. Source data are provided as a Source Data file.

Supplementary Note 3. Branch length comparison at the phylum-level

To assess whether the patterns observed for the entire bilaterian dataset are consistent across lower taxonomic levels, we selected taxa that comprised different life-history categories and analysed them separately. Multiple life histories were observed in five phyla, and multiple locomotory capacities in four, but they overlapped only in Arthropoda, where branch lengths mirrored the overall bilaterian pattern almost perfectly: shortest in microparasites and free-living, followed by parasitoids, ectoparasites, and endoparasites (Supplementary Figure 5). Notably, the value range was very wide among the arthropods in the free-living category. Similarly, branch lengths were the lowest in the High locomotory capacity category, followed by the Intermediate and Low categories in Arthropoda

(Supplementary Figure 6). Intriguingly, the impact of locomotory capacity categorisation outweighed the impact of life history categorisation in the *lmekin* multilevel phylogenetic regression analysis (Figure 3 in the main manuscript).

In Annelida, branch lengths were significantly higher in microparasitic than in free-living species, but the number of microparasitic samples was low (5) (Supplementary Figure 5). In the Chordata, microparasitic species did not differ significantly from the free-living, but in the locomotory capacity categorisation, branch lengths were more than twice as large in the Low group as in the High and Intermediate groups (Supplementary Figure 6). In Platyhelminthes, branches were the longest in endoparasites, followed by ectoparasites, and more than twice shorter in free-living species. Nematoda was a major exception, with endoparasites exhibiting almost identical branch lengths to the free-living species (but the power of this analysis was weak; Supplementary figure 7). A minor, albeit intriguing, exception was Mollusca: in agreement with the working hypothesis, branches were the shortest in the High category, but the Intermediate locomotory capacity category had significantly longer branches than the Low locomotory capacity category. This pattern was replicated by the classes Bivalvia and Gastropoda, but not by Cephalopoda (see Supplementary Note 4).

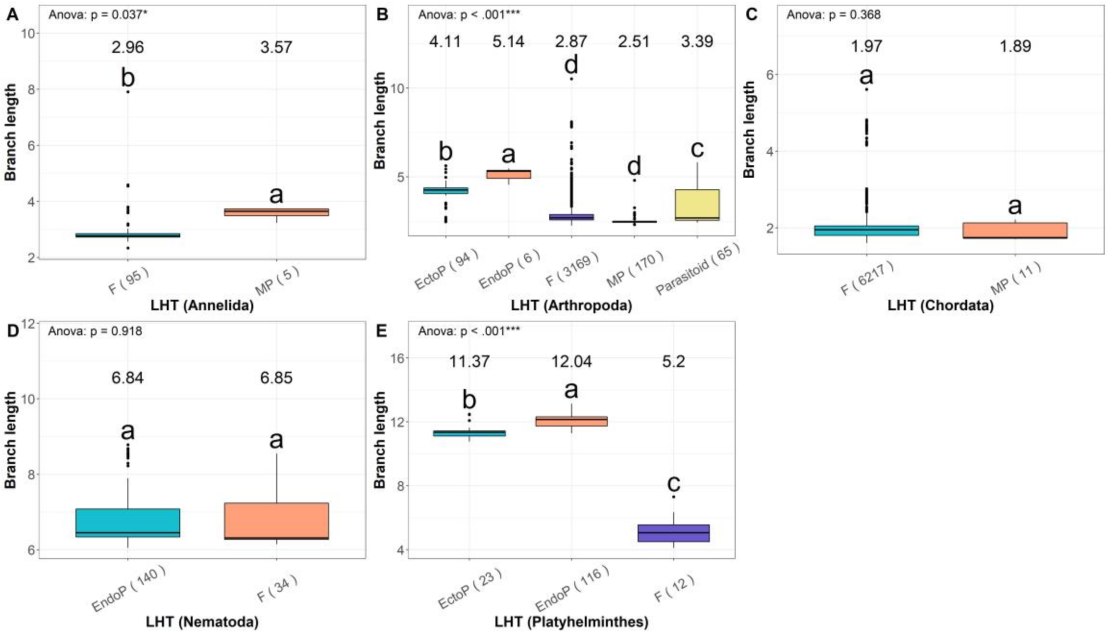

Supplementary Figure 5. Comparison of average branch lengths in different life history (LHT) categories at the phylum level. Average branch length values are shown above the boxplots. F is free-living, EndoP is endoparasites, EctoP is ectoparasites, and MP is micropredators. Average branch length values are shown above the boxplots. PGLS ANOVA results are shown in the upper left corner. Different letters above the boxplots indicate statistically significant differences (p<0.05). The number of species included in the analysis is shown next to the category name (x-axis). Source data are provided as a Source Data file.

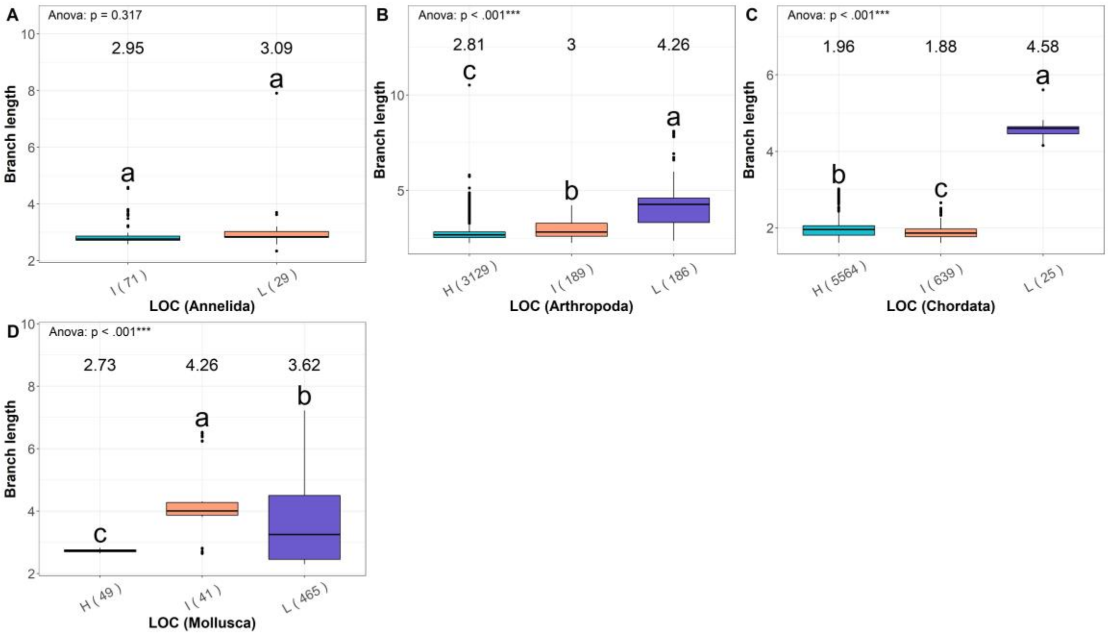

Supplementary Figure 6. Comparison of average branch lengths in different locomotory capacity (LOC) categories at the phylum level. H is High, I is Intermediate, and L is Low (locomotory capacity). For other details see Supplementary Figure 5. Source data are provided as a Source Data file.

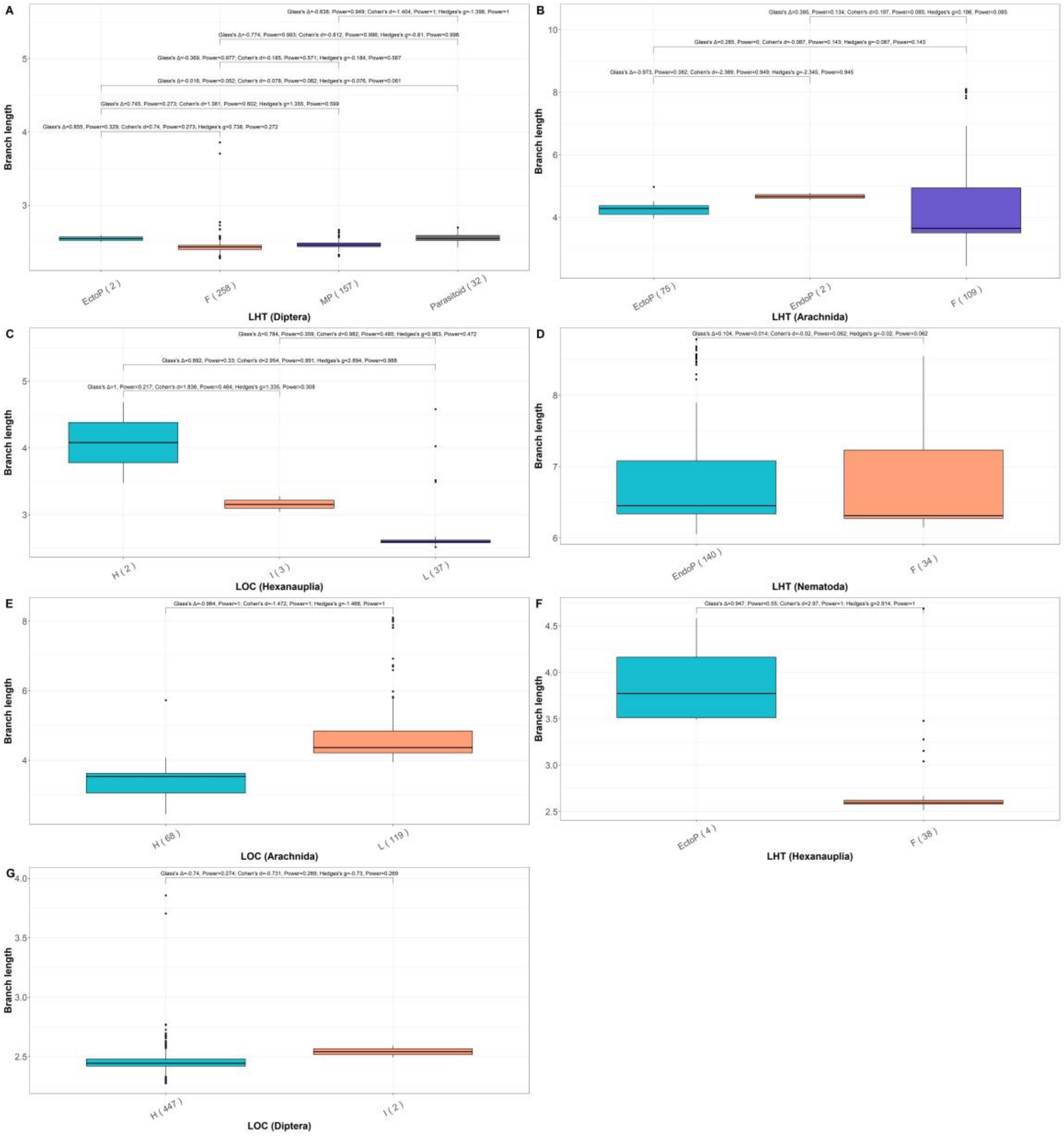

Supplementary Figure 7. The effect sizes and statistical power analyses for pairwise group comparisons of taxa that exhibited branch length patterns that were in disagreement with our working hypotheses. In the life-history categorisation (LHT), F is free-living, EndoP is endoparasites, EctoP is ectoparasites, and MP is micropredators. In the locomotory capacity (LOC) categorisation, H is High, I is Intermediate, and L is Low. Categories with only one species were removed from the analyses (MP in Arachnida, and Low LOC in Diptera). Nematoda only had the Low LOC category, so we could not conduct locomotory capacity analysis. Three different effect size measures were used. Cohen's d should be used when the two groups have similar standard deviations and the same sample size that is >20. Glass's Δ should be used when the two groups' standard deviations are different. Hedges's g should be used when the two groups have similar standard deviations and different sample sizes, or

both groups have a sample size <20 per group. Cohen's d values can be interpreted in the following way: d<0.2 = small effect size, 0.2<d<0.5 = medium, 0.5<d<0.8 = large, and d>0.8 = very large. Statistical power analyses were conducted for each effect size measure using the pwr.t2n.test method in the R package 'pwr'. Power refers to the probability of detecting a statistically significant effect if one exists, where the commonly accepted cut-off value for a good statistical power is ≥ 0.8. Source data are provided as a Source Data file.

Supplementary Note 4. Branch length comparison at the class level

At the class level, there was more than one life history category in nine classes and more than one locomotory capacity category in twelve classes, but many analyses were weakened by a very small number of samples (<10) (Supplementary Figures 8 and 9). Insecta was among the very few classes that comprised multiple categories in both tested variables; branch lengths in the LHT categorisation were microparasites < free-living < ectoparasites ≤ parasitoids (nonsignificantly different); as regards the locomotory capacity categorisation, branch lengths followed the bilaterian pattern: High < Intermediate < Low. In Hexanauplia, ectoparasites had longer branches than free-living, while in Clitellata and Mammalia, microparasites had longer branches than free-living, but the numbers of samples were low. Both Nematoda classes (Chromadorea and Enoplea) were in disagreement with the bilaterian patterns and our hypotheses, with endoparasites and free-living species exhibiting nonsignificantly different branch lengths. Arachnida was also a major apparent exception: branch lengths were nonsignificantly lower in ectoparasites (4.25) than in free-living species (4.36). The numbers of endoparasites (2) and microparasites (1) were too low to draw any conclusions. As this lineage was a major outlier in relation to the overall pattern, and free-living lineages had a wide distribution, we analysed it in more detail.

Four orders exhibited average branch lengths higher than strictly ectoparasitic Ixodida (4.24): Mesostigmata (a mix of life histories), Sarcoptiformes (a mix of free-living and ectoparasitic), Pseudoscorpiones (free-living), Trombidiformes (free-living). Pseudoscorpiones aside (represented by only one species in the dataset), the remaining three orders comprise mites with low locomotory capacity, similar to that exhibited by parasitic mites – ticks (Supplementary Table 5). Also, the classification of Trombidiformes was difficult, as their life history borders between free-living and parasitoid. For example, larval forms of some

lineages, such as *Leptotrombidium*, feed on the skin of mammals. Therefore, their low locomotory capacity and parasitoid-adjacent life history may explain this outlier with respect to our working hypotheses. In agreement with this, branches were significantly longer in the Low LOC group than in the High LOC group in Arachnida (Supplementary Figure 9C).

As regards locomotory capacity, High and Low categories were included only in Arachnida and three Arthropoda classes: Insecta, Malacostraca, and Hexanuplia. In Arachnida, Insecta and Malacostraca, the branch lengths were in agreement with our predictions: shortest in the High category, followed by the Intermediate category (where available), and the longest in the Low category. Hexanauplia were an outlier, with the Low category exhibiting the shortest branches, but there were only 2 species in the High category (both Harpacticoida). Also, the order Sessilia of the Hexanauplia class was previously recognised as a major outlier among crustaceans: despite its sessile lifestyle, it appears to exhibit very strong purifying selection pressures [25]. This indicates that Sessilia is a major outlier not only among crustaceans but also among all bilaterian animals. In other taxa, there were only two combinations, Intermediate/High and Intermediate/Low; and the results were rather inconsistent.

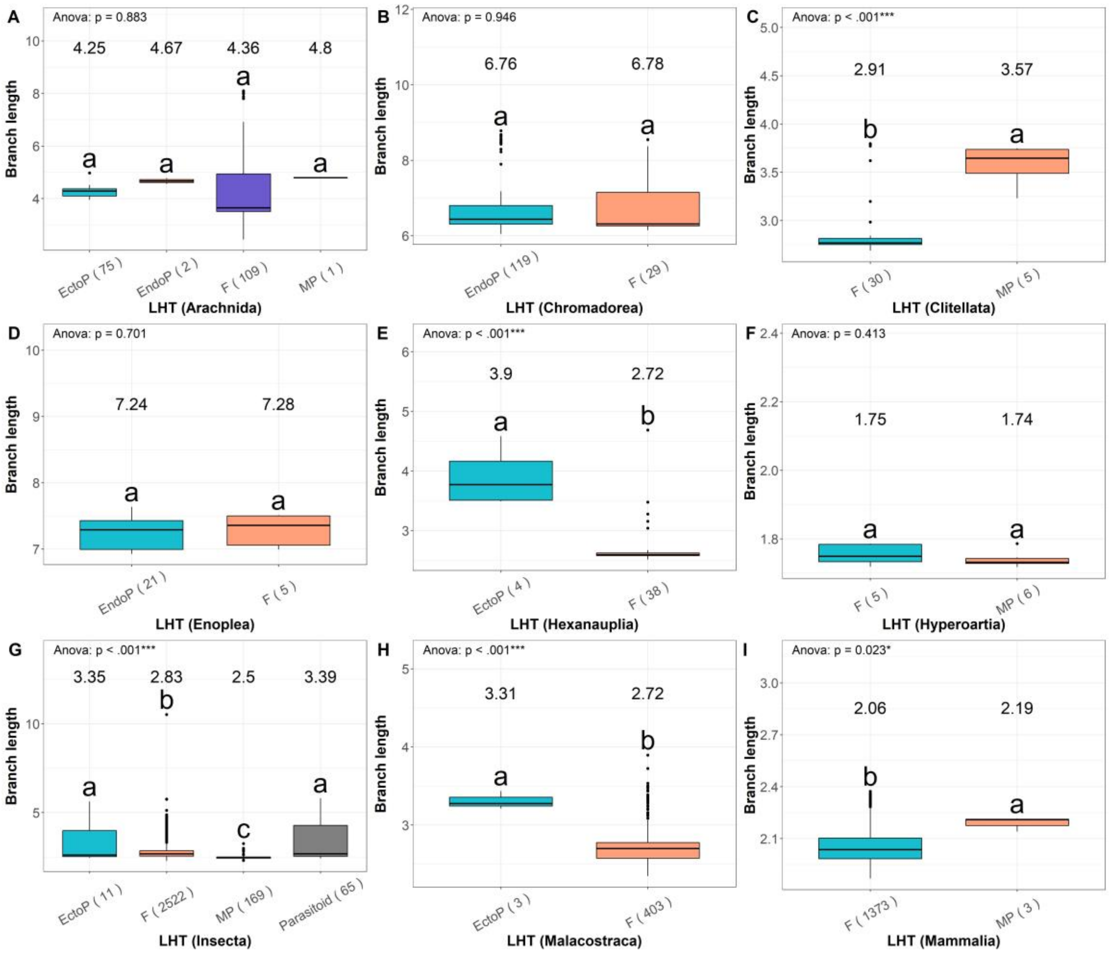

Supplementary Figure 8. Comparison of average branch lengths in different life history (LHT) categories at the class level. Average branch length values are shown above the boxplots. F is free-living, EndoP is endoparasites, EctoP is ectoparasites, and MP is micropredators. Average branch length values are shown above the boxplots. PGLS ANOVA results are shown in the upper left corner. Different letters above the boxplots indicate statistically significant differences (p<0.05). The number of species included in the analysis is shown next to the category name (x-axis). Hexanauplia is currently accepted as Thecostraca. Source data are provided as a Source Data file.

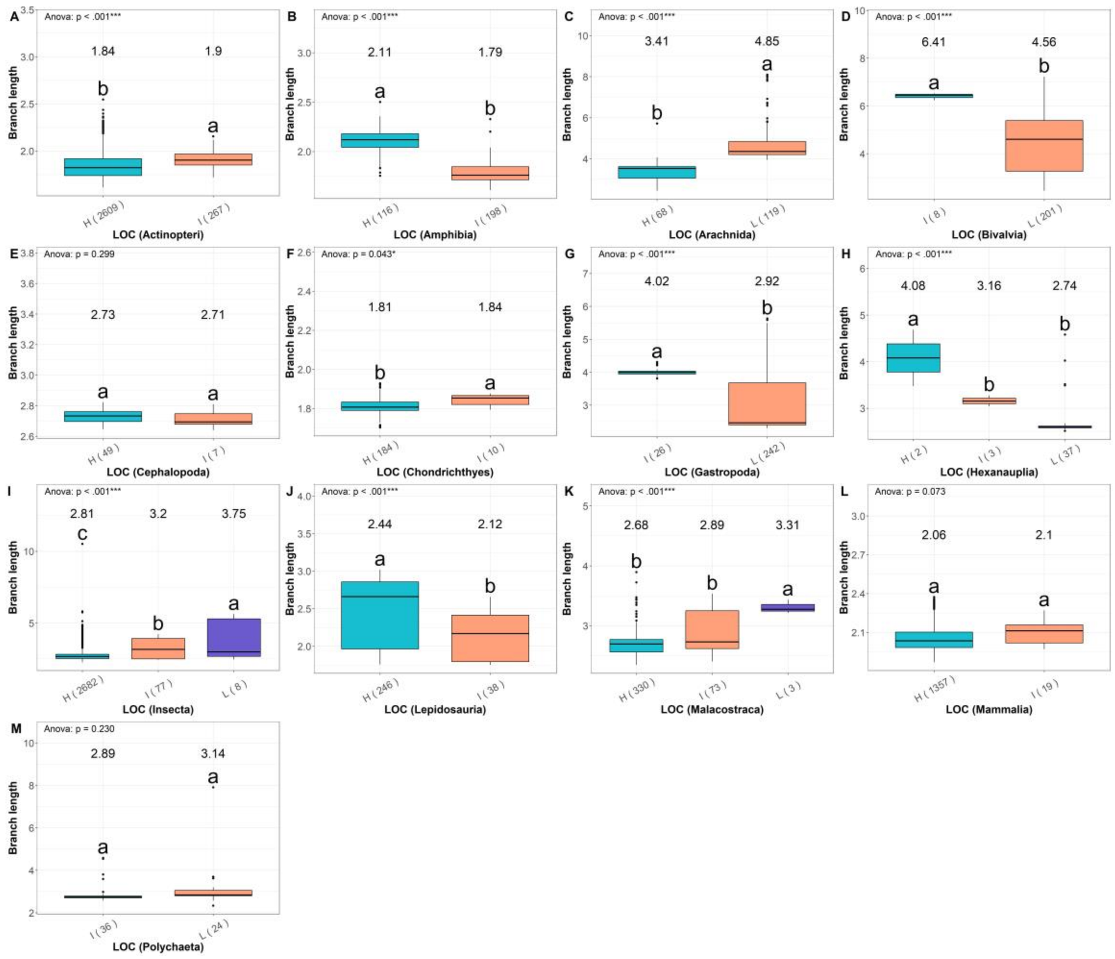

Supplementary Figure 9. Comparison of average branch lengths in different locomotory capacity (LOC) categories at the class level. H is high, I is Intermediate, and L is low (locomotory capacity). For other details see Supplementary Figure 8. Source data are provided as a Source Data file.

Supplementary Table 5. Branch lengths (brl) in the orders of Arachnida. No. is the number of species included in the analysis, brl is the average branch length, LHT is life history (F is free-living, EndoP is endoparasites, EctoP is ectoparasites, and MP is micropredators), and LOC is the locomotory capacity (H is High, I is Intermediate, and L is Low). Source data are provided as a Source Data file.

Order	No.	brl	LHT	LOC
Trombidiformes	23	6.86	F	L

Pseudoscorpiones	1	5.72	F	H
Sarcoptiformes	13	4.89	F+EctoP	L
Mesostigmata	10	4.59	F+EctoP+EndoP+MP	L
Ixodida	73	4.24	EctoP	L
Araneae	49	3.61	F	H
Ricinulei	4	3.02	F	H
Amblypygi	1	2.85	F	H
Opiliones	3	2.78	F	H
Scorpiones	8	2.60	F	H
Solifugae	2	2.57	F	H

Supplementary Note 5. Branch length comparison at the order level

As regards the order level, many analyses were weakened by a low number of samples for some categories (Supplementary Figures 7, 10, and 11). In Diptera (Arthropoda: Insecta), branches were the longest in parasitoids, followed by ectoparasites, micropredators, and finally free-living, but all values were similar, so none of the differences was statistically significant. There were only three ectoparasitic species, which weakened the statistical analyses. Notably, all three belong to the superfamily Hippoboscoidea: 2 Streblidae (bat flies) and 1 Hippoboscidae (*Melophagus ovinus* or sheep ked). Both families exhibit remarkably high locomotory capacity in comparison to most other ectoparasites, aside from fleas. In many ways, their classification was difficult, because they resemble micropredators in their lifestyle. In addition, sheep ked is an exception within Hippoboscidae, for being the only member that has completely lost its wings [26], so the loss of high locomotory capacity in this lineage appears to be of relatively recent evolutionary origin. Therefore, locomotory capacity appears to be a suitable explanation for this outlier as well. In Rhabditida (Nematoda: Chromadorea), endoparasites exhibited longer branches than free-living, but the difference was nonsignificant, likely due to the wide distribution of data in endoparasites. There were no significant differences between the free-living and parasitoids in Hymenoptera, and free-living and microparasites in Petromyzontiformes and Hirudinida. In Chiroptera, microparasites (only three species) had non-significantly longer branches than free-living, but the opposite pattern was observed in Hemiptera (significantly different).

As regards the locomotory capacity, most orders merely had High and Intermediate categories, or the numbers of samples were too small to draw any conclusions (<3). In most cases, differences were small and nonsignificant.

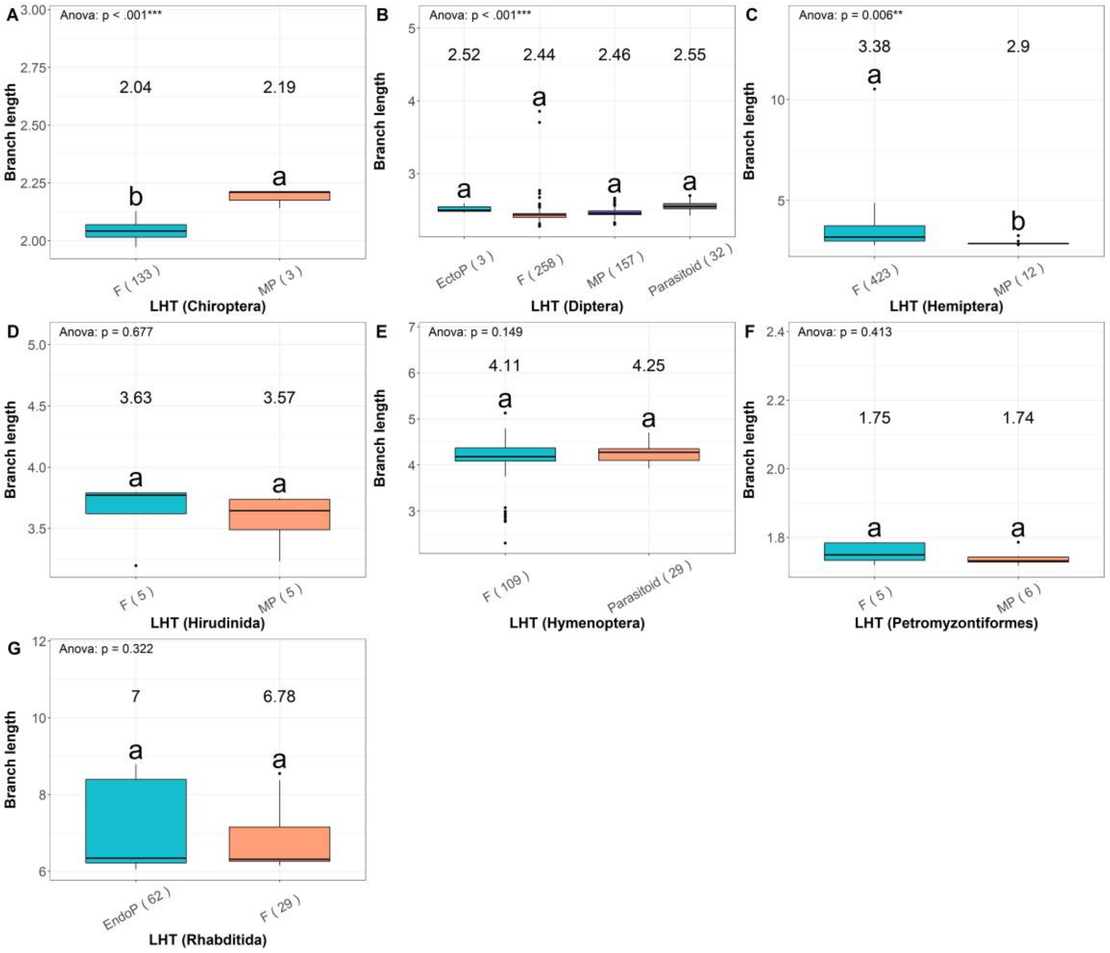

Supplementary Figure 10. The comparison of average branch lengths in different life history categories (LHT) at the order level. Average branch length values are shown above the boxplots. F is free-living, EndoP is endoparasites, EctoP is ectoparasites, and MP is micropredators. Average branch length values are shown above the boxplots. PGLS ANOVA results are shown in the upper left corner. Different letters above the boxplots indicate statistically significant differences (p<0.05). The number of species included in the analysis is shown next to the category name (x-axis). Source data are provided as a Source Data file.

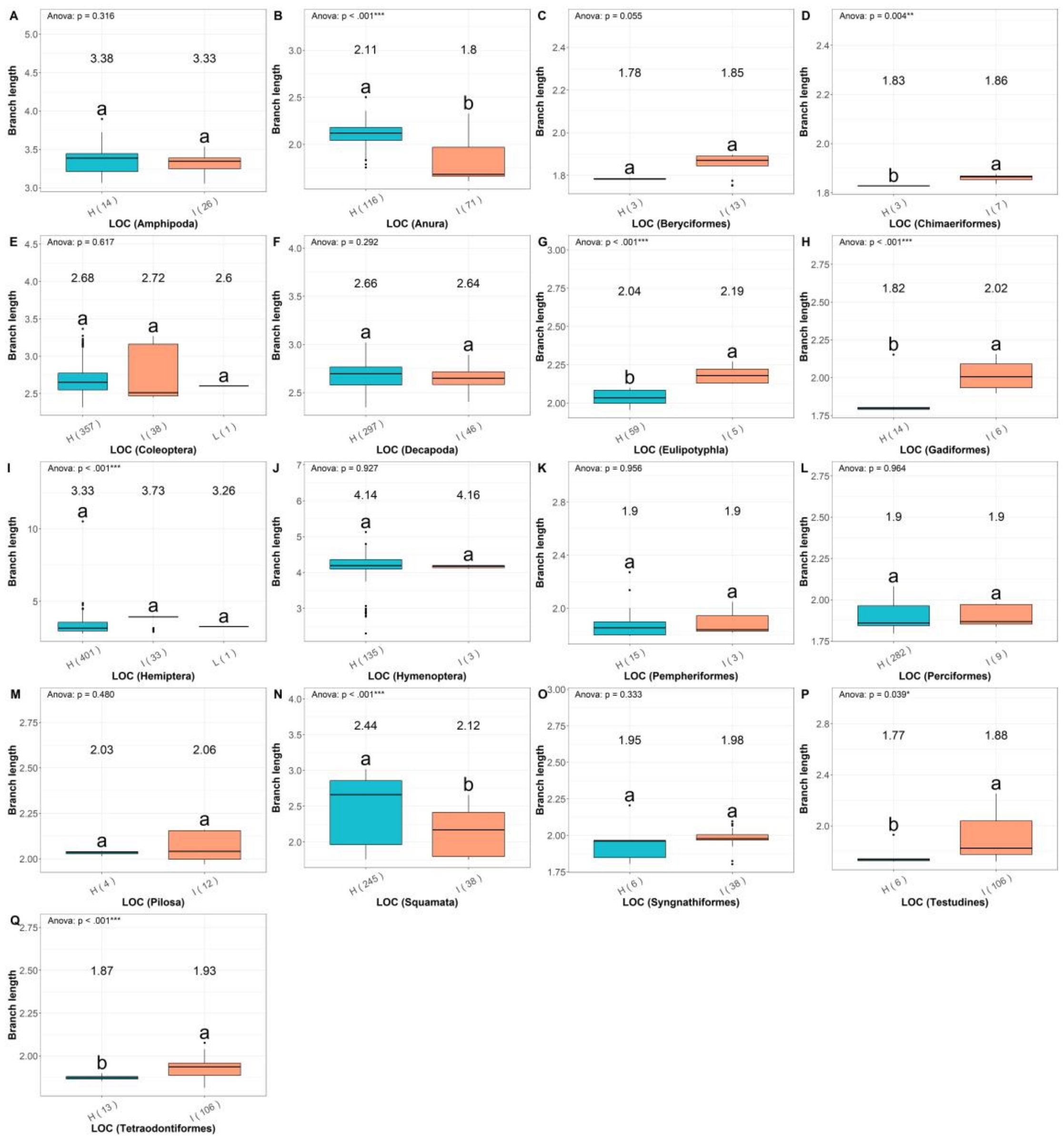

Supplementary Figure 11. The comparison of average branch lengths in different locomotory capacity categories (LOC) at the order level. H is High, I is Intermediate, and L is Low (locomotory capacity). For other details see Supplementary Figure 10. Source data are provided as a Source Data file.

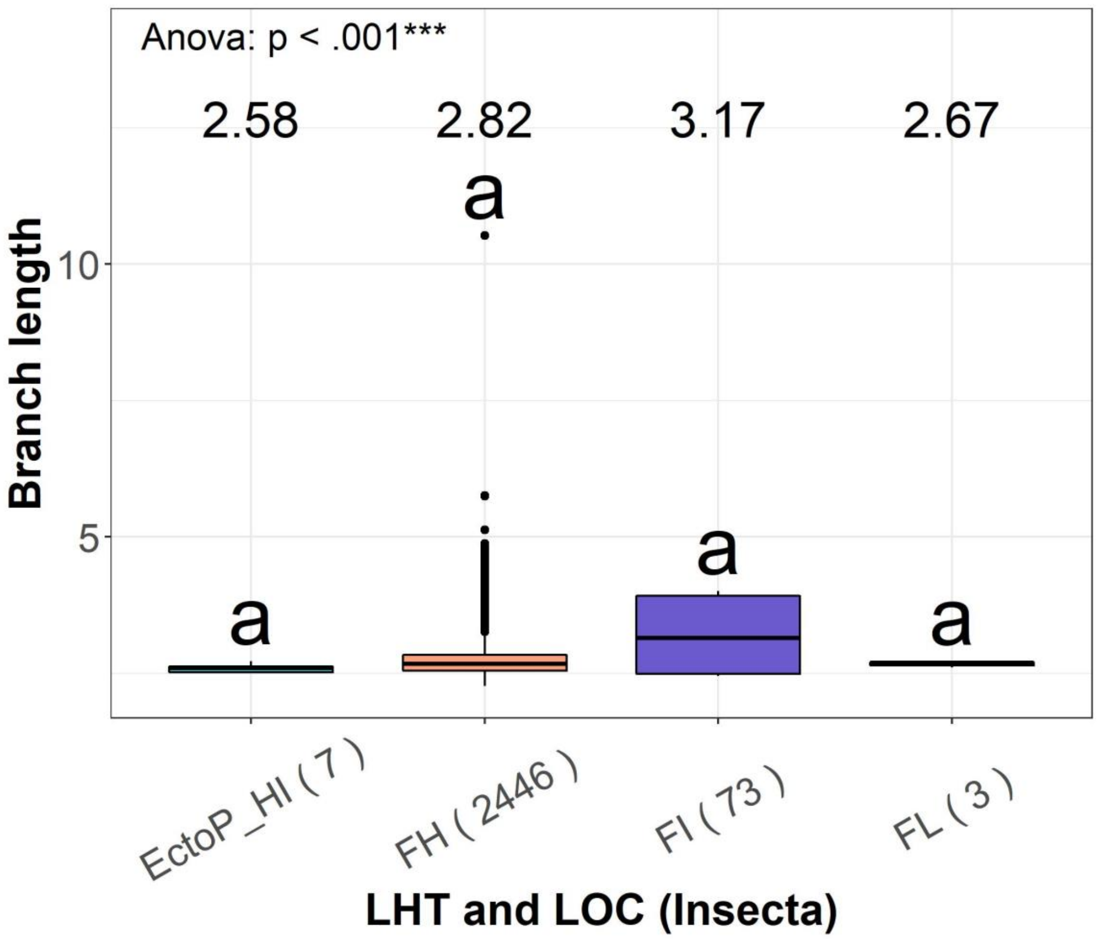

Supplementary Figure 12. Branch lengths in ectoparasites classified into High and Intermediate locomotory capacity (LOC) categories (EctoP_HI) vs. the free-living Insecta divided along the LOC categorisation. Average branch length values are shown above the boxplots. FH: free-living lineages with high LOC, FI: free-living with Intermediate LOC, FL: free-living with low LOC. Average branch length values are shown above the boxplots. PGLS ANOVA results are shown in the upper left corner. Different letters above the boxplots indicate statistically significant differences (p<0.05). The number of species included in the analysis is shown next to the category name (x-axis). Source data are provided as a Source Data file.

Supplementary Note 6. Single-gene results

We further assessed whether single-gene topologies may produce different results by constructing topology-constrained single-gene trees for all 12 PCGs using FastTree, extracting branch lengths and assessing correlation with the main tree (produced using concatenated genes - AAs_C50). Branch lengths of single-gene trees generally had a

relatively high correlation of over 70%, with the highest values exhibited by *cox1* and *cox3* (94-95%). The exceptions were *nad2*, *nad4L*, and *nad6*, which had low correlations with the main tree: 23%, 41%, and 18%, respectively (Supplementary Data 1: Worksheet 4). As *nad2* is the only relatively large gene among the three outlier genes (≈1000 bp), we conducted pairwise comparative analyses using only the *nad2* branch length data. In the main aspects, the results were congruent with the overall dataset (Supplementary Figure 13).

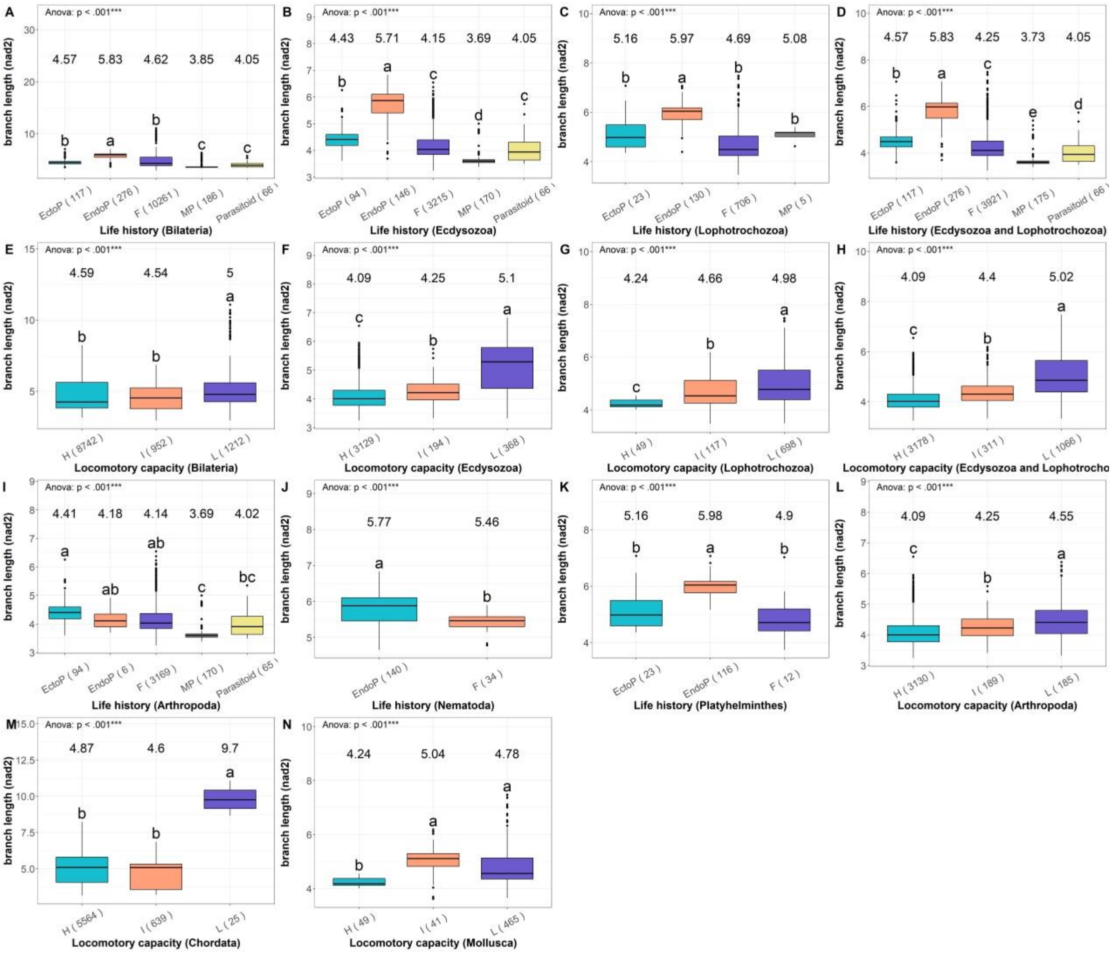

Supplementary Figure 13. ***nad2*** **tree branch length comparison between different life history categories and locomotory capacity categories.** Average branch length values are shown above the boxplots. In the life-history categorisation, F is free-living, EndoP is endoparasitic, EctoP is ectoparasitic, and MP is micropredatory. In the locomotory capacity categorisation, H is high, I is Intermediate, and L is Low. PGLS ANOVA results are shown in the upper left corner. Different letters above the boxplots indicate statistically significant differences (p<0.05). The number of species included in the analysis is shown next to the

category name (x-axis). Source data are provided as a Source Data file.

Supplementary Table 6. Phylogeny-corrected lmekin and brms regression analyses with branch length as the dependent variable and dataset divided along different life history lines as the independent variable. R^2 evaluates how much a variable can explain the variance of the dependent variable (calculated via univariate regression). aR^2 is the adjusted R^2 value. F is free-living, EndoP is endoparasitic, EctoP is ectoparasitic, and MP is micropredatory. In the *brms* analyses, Estimates (Est) are given with the standard deviation of the posterior distribution (Error) and lower and upper 95% credible intervals (l and u respectively); R^2 values are also given with the standard deviation (R^2 Error) and indicate lower and upper boundaries of the 95% credible interval (Q2.5 and Q97.5 respectively).

**Dataset**	**LMEKIN**		**BRMS**							
	**R^2**	**aR^2**	**Est**	**Error**	**l**	**u**	**R^2**	**R^2 Err**	**Q2.5**	**Q97.5**
EndoP vs. EctoP vs. (F+MP) vs. parasitoid	0.4251	0.4516	1.63	0.09	1.46	1.79	0.4247	0.0051	0.4146	0.4346
EndoP vs. EctoP vs. (F+MP+parasitoid)	0.4183	0.4443	1.62	0.08	1.46	1.80	0.4179	0.0055	0.4073	0.4286
(EndoP+EctoP) vs (F+MP) vs. parasitoid	0.4056	0.4308	3.39	0.04	3.31	3.47	0.4050	0.0057	0.3937	0.4161
(EndoP+EctoP) vs. (F+MP+parasitoid)	0.3987	0.4235	3.38	0.04	3.31	3.46	0.398	0.0055	0.3867	0.4085

Supplementary Table 7. BRMS multilevel regression analyses with branch length as the dependent variable. Every parameter is summarized using the mean (Est.), standard deviation (Error) of the posterior distribution, as well as two-sided 95% Credible Intervals (l-95% and u-95%) based on quantiles. R^2 evaluates how much a variable can explain the variance of the dependent variable (calculated via univariate regression). The random effect was not included in any of the analyses. Ind var = Independent variable, ncp = not corrected for phylogeny. LOC is locomotory capacity, and LHT is life history. LHT×LOC tests the interaction between the two variables. N/A denotes not available.

Dataset	Ind var	Est	Est Error	l-95%	u-95%	R^2	R^2 Error	Q2.5	Q97.5
Bilateria	LHT	1.62	0.08	1.46	1.78	0.4259	0.0053	0.4153	0.4363
Bilateria	LOC	-0.02	0.03	-0.07	0.03	0.3687	0.0058	0.3576	0.3800
Bilateria	LHT+LOC	1.55	0.08	1.40	1.70	0.5275	0.0046	0.5184	0.5367
Bilateria	LHT+LOC(ncp)	1.55	0.08	1.40	1.70	0.5277	0.0045	0.5191	0.5364
Bilateria	Null model	N/A	N/A	N/A	N/A	2.16E-30	4.65E-30	1.53E-34	1.49E-29
Ecdysozoa	LHT	1.94	0.09	1.76	2.12	0.4677	0.0087	0.4504	0.4844
Ecdysozoa	LOC	0.24	0.05	0.14	0.34	0.5087	0.0077	0.4935	0.5238
Lophotrochozoa	LHT	0.02	0.12	-0.21	0.25	0.7316	0.0083	0.7138	0.7469
Lophotrochozoa	LOC	0.35	0.16	0.04	0.67	0.0690	0.0157	0.0404	0.0995
Ecdysozoa+Lophotrochozoa	LHT	1.54	0.07	1.41	1.68	0.5644	0.0063	0.5518	0.5763
Ecdysozoa+Lophotrochozoa	LOC	0.30	0.05	0.20	0.39	0.3697	0.0090	0.3513	0.3875
Bilateria	LHT+LOC+LHT×LOC	570.28	197.02	146.97	875.30	0.5230	0.0045	0.5202	0.5381
F	Low LOC	-0.02	0.03	-0.08	0.04	0.1868	0.0062	0.1749	0.1991
F	Null-model	N/A	N/A	N/A	N/A	1.93E-30	3.89E-30	3.87E-34	1.32E-29
Low LOC	LHT	0.94	0.08	0.78	1.08	0.5209	0.0135	0.4939	0.5467
Low LOC	Null-model	N/A	N/A	N/A	N/A	2.12E-31	4.6E-31	2.28E-35	1.46E-30

Supplementary Note 7. Selection analyses

The bilaterian dataset comprised three mitochondrial genetic codes: invertebrate ("Table 5"), echinoderm and flatworm ("Table 9"), and vertebrate ("Table 2"). As vertebrates were almost all free-living and mostly assigned to High and Intermediate locomotory capacity categories, we excluded them from the dataset. Among the genetic code table 5 lineages, we selected all endoparasites, ectoparasites, and parasitoids, and compared each to a different selection of free-living lineages. We also compared subsets of Low and High locomotory capacity category species. As Platyhelminthes employ a different genetic code (code table 9), we tested them separately. To improve the resolution, we used the dataset comprising all available flatworm mitogenomes (223 species) from our recent study of patterns of mitogenomic evolution in flatworms [27]. We further tested Nematoda separately, because they were a major outlier in relation to our hypotheses (code table 5; two LHT categories: EndoP and F). As Arthropoda comprised the most complex evolutionary scenarios, with multiple independent origins of parasitism and high locomotory capacity, we also tested them separately. As these analyses are computationally demanding, we selected subsets of free-living lineages for the "Table 5" dataset. For LHT comparisons we selected sister lineages to all parasitic clades, and for LOC comparisons we selected sister lineages to species classified into the Low locomotory capacity category. Due to the uneven distribution of both traits, in many cases it was not possible to select a closely related lineage. This caused low levels of homology among fast-evolving genes, thus weakening the resolution of our analyses, so results should be interpreted with this limitation in mind. As Arthropoda comprised the most complex evolutionary scenarios, with multiple independent origins of parasitism and high locomotory capacity, we also tested them separately using selected lineages. As the Table 5 and 9 codes exhibit only minimal differences, for the comparisons of ectoparasites with low and high locomotory capacity (few species), we merged the "Table 5" and "Table 9" datasets to improve the resolution.

While most results are in agreement with the observed branch length patterns and our hypotheses, there were also several exceptions. A minor unexpected result was that RELAX found evidence for intensified/neutral purifying selection pressure in endoparasites compared to ectoparasites in Table 5 and Arthropoda datasets respectively, whereas branch patterns would make us expect relaxed purifying selection in endoparasites. Notably, the average omega values were higher in endoparasites in the arthropod dataset, indicating relaxed purifying selection. Also, these analyses encountered convergence issues, so these may be artefacts, possibly caused by high levels of substitution saturation in rapidly evolving macroparasitic lineages. To further test this, we selected only the leaf branches in the Arthropoda dataset and rerun the analysis. Indeed, in this analysis,

endoparasites exhibited relaxed selection pressure compared to ectoparasites. This indicates that these analyses are sensitive to the inclusion/exclusion of inner tree branches.

Low locomotory capacity lineages exhibited neutral purifying selection pressures compared to the High category when we tested a broad range of selected lineages employing the Table 5 code. However, apart from Cephalopoda, all other lineages with High locomotory capacity belonged to Arthropoda, so the distribution of High LOC lineages was highly uneven. To address this, we separately tested only the Arthropoda sub-dataset (selected Low and High LOC lineages). Using this dataset, we found evidence of significantly (albeit weakly) relaxed purifying selection pressures in the Low locomotory capacity category. We tested the same hypothesis using only the ectoparasitic dataset, but the analyses produced an inconsistent pattern, comprising intensified (all ectoparasites) and neutral (only arthropod ectoparasites) datasets. Although these results partially contradict branch length patterns and our hypotheses, the results inferred using the Arthropod dataset indicate that these may be artefacts caused by highly divergent sequences, as less phylogenetically divergent datasets are expected to produce more reliable results due to higher levels of (recognisable) homology and thus improved codon alignment.

As regards directional selection, in most cases, there were signals of significant directional selection in both parasitic and free-living lineages, and Low and High locomotory capacity lineages. Exceptions were parasitoids and flatworms, where there was no significant evidence for directional selection in free-living lineages. Also, there was no evidence for directional selection in endoparasitic arthropods, but the dataset was small (6 species).

We wish to emphasise once again that these results should be interpreted with caution due to various sources of noise, already discussed in this section, comprising the violations of assumptions of the parameter-rich algorithms employed, artefactual relationships in phylogenetic reconstructions, pruning of many lineages, as well as high levels of mutational saturation of data and difficult inference of homology in a dataset comprising phylogenetically highly diverse lineages.

Supplementary Table 8. RELAX and BUSTED selection pressure tests. RELAX tests the hypothesis that purifying selection pressure is relaxed in the test branches (left) vs. the background branches (right). BUSTED tests the hypothesis that episodic diversifying positive selection occurred on test and/or background branches. In brackets next to group names are the numbers of species included in the dataset. LOC is the locomotory capacity categorisation. K is a measure of selection pressure, where values <1 indicate the relaxed purifying selection, >1 indicate the intensified purifying selection, and 1 indicates neutral selection. * indicates convergence issues. RELAX statistically tests (LRT test) whether the K value significantly (p<0.05) differs from 1 (p-values column, where p = 0 is shown as p < 0.0001). # indicates that code Table 5 and 9 species were all included in the same dataset. For the Platyhelminthes dataset, we used an extended dataset comprising all species available in the GenBank. Arth refers to the Arthropoda dataset, and "tips" means that only branch tips were selected in the arthropod tree.

		**RELAX**					**BUSTED**	
**Dataset**	**Compared groups**	**Selection**	**K**	**p**	**ω test**	**ω ref**	**Test**	**Background**
Code table 5	EndoP (160) vs. F (61)	relaxation*	0.96	<0.0001	0.049	0.029	<0.0001	<0.0001
	EndoP (160) vs. EctoP (94)	Intensification*	1.08	<0.0001	0.071	0.091	<0.0001	<0.0001
	EctoP (94) vs. F (84)	relaxation	0.930	<0.0001	0.093	0.080	<0.0001	<0.0001
Parasitoid	Parasitoid (66) vs. F (66)	relaxation	0.640	<0.0001	0.079	0.044	<0.0001	0.5000
Platyhelminthes	EndoP (152) vs. F (30)	relaxation	0.84	<0.0001	0.083	0.060	<0.0001	0.5000
	EctoP (41) vs. F (30)	relaxation	0.87	<0.0001	0.121	0.079	0.0002	0.5000
	EndoP (152) vs. EctoP (41)	relaxation	0.90	<0.0001	0.082	0.061	<0.0001	<0.0001
	EndoP+EctoP (193) vs. F (30)	relaxation	0.93	<0.0001	0.0708	0.0552	<0.0001	0.5000
Nematoda	EndoP (140) vs. F (34)	relaxation	0.76	<0.0001	0.066	0.038	<0.0001	0.0239
Arthropoda	EndoP (6) vs. EctoP (94)	neutral*	1.09	1.0000	0.155	0.079	0.5000	<0.0001
Arth_tips	EndoP (6) vs. EctoP (94)	relaxation*	0.76	<0.0001	0.096	0.058		

EctoP[#]	Low (110) vs. High (6) LOC	intensification	1.070	0.0001	0.093	0.119	<0.0001	<0.0001
EctoP_Arth	Low (87) vs. High (6) LOC	neutral	1.00	1.0000	0.088	0.113	<0.0001	<0.0001
LOC table 5	Low (191) vs. High (233) LOC	neutral	1.00	1.0000	0.082	0.085	<0.0001	<0.0001
LOC Arthropoda	Low (39) vs. High (176) LOC	relaxation	0.99	0.0038	0.106	0.101	<0.0001	<0.0001

Supplementary Discussion

Parasitoids had slightly but statistically significantly elevated evolutionary rates compared to free-living organisms, but they exhibited far shorter branches than macroparasites. Therefore, we hypothesise that strong selection for high locomotory capacity limits the strong relaxation of purifying selection pressures, observed in most other macroparasites. This may explain inconsistent results in previous studies of parasitoid wasps [6,16]. We should note that selection analyses found signals of relaxed purifying selection pressures and elevated rates of directional selection in parasitoid organisms (compared to free-living).

Many variables have been associated with mitogenomic evolution, and the parsing of their impacts is further complicated by their interrelatedness [25]. It is beyond the scope of this study to discuss all of them in detail, but we will try to address the major ones succinctly.

The effects of some variables appear to be lineage-specific. This comprises locomotory capacity, which is limited (albeit less so than in parasites) in free-living Platyhelminthes, but branch lengths are almost twice the length in parasitic compared to the free-living lineages, so selection for locomotory capacity does not appear to be the major factor behind these elevated evolutionary rates. Notably, disproportionately long branches in parasitic Platyhelminthes are largely attributable to a very long stem branch [5] and our analyses indicate that they may be undergoing elevated levels of directional evolution. An additional source of noise in our dataset might be the fact that some macroparasitic lineages go through free-living nonadult developmental stages that possess a non-rudimentary locomotory capacity, such as some parasitic copepods [37]. In vertebrates, body size/mass and metabolic rate calibrate the (mitochondrial and nuclear) molecular clock (smaller animals = faster evolution) [28–30], but there is no evidence for this in invertebrates [31,32]. Indeed, some parasitic flatworms, such as some cestodes, are orders of magnitude larger than free-living ones, and yet they exhibit several times faster evolutionary rates.

Generation time appears to have effects in both vertebrates and invertebrates: organisms with shorter generation times have a greater number of germ-cell divisions per year, and thus more replication errors per time unit, leading to faster mutation rates [28,33]. A closely related variable, longevity (or lifespan), was also associated with mitogenomic evolution. The hypothesis proposes that long-lived animals may have adapted to an increased lifespan by evolving macromolecular components more resistant to oxidative damage, thus reducing their evolutionary rates [34–36]. This hypothesis has been thoroughly tested in mammals, and several studies also found support for it in

invertebrates [33,35,37,38]. However, multiple studies reported that there are exceptions from the expected negative relationship between longevity and/or generation time and molecular clock rate [27,34,39–42]. While parasitic lineages do have shorter generation times than their vertebrate hosts [43], this may not be a rule in invertebrates [44]. For example, many parasitic flatworms and nematodes have complex life cycles that require multiple hosts and maturation stages, which results in rather long generation times [43]. As many small invertebrates have very short generation times [33,45], it is unclear whether there is a significant difference in generation times between closely related parasitic and free-living invertebrate lineages. Also, multiple parasitic lineages (sometimes) exhibit rather long life spans, such as parasites in paratenic hosts. It would be interesting to study the impact of this variable on mitogenomic evolution in parasitic lineages, but the limited availability of data may be a confounding factor.

The effective population size (N_e) is positively correlated with the strength of purifying selection, so species with lower N_e should exhibit higher rates of mitochondrial evolution [46]. This also implies that the speciation rate may be positively correlated with the evolutionary rate via the founder effect [47]. The evidence for the putative correlation between the N_e and mitochondrial evolution remains elusive: a case study found a negative correlation [48], but several studies of much larger datasets found weak evidence [49], or no evidence at all [25,27,50]. A study argued that this may be due to large differences in mutation rates [41], so the impact of N_e remains debatable. It should be noted that molecular *Ne* estimates are sensitive to a number of methodological and life history parameters [51,52], so *Ne* inference can be rather difficult and error-prone. Most importantly, the *Ne* is highly likely to fluctuate strongly throughout the evolutionary history of any lineage [53], so the current *Ne* values may be completely decoupled from the root to tip branch lengths of the lineage.

Several studies found support for the hypothesis that thermic habitat affects mitochondrial evolution [54,55] and that evolutionary rates differ between endotherms and ectotherms [39]. The ‘functional constraints’ hypothesis proposes that variations in the thermic environment may restrict physiologically acceptable amino acid substitutions, which implies that thermally stable endotherms should have a higher rate of sequence evolution than thermally variable ectotherms [39,56]. However, several studies found that the impact of thermic habitat is very weak or inconsistent [27,39,56,57].

Mutation pressure is presumed to be positively correlated with the metabolic rate: a high metabolic rate increases the overall time that the mitochondrial H-strand spends in the mutagenic single-strand state (replication and transcription) and increases the production of mutagenic reactive oxygen species [28,32,39,58–60]. However, studies failed to find evidence for the association of metabolic and mutational rates in isopods [42], crustaceans [25], and across the Metazoa [32].

Another important variable may be variability in mtDNA replication and repair mechanisms: lower fidelity = higher evolution [33,61–64]. However, multiple independent origins of parasitism in Bilateria [24] suggest that it is statistically highly unlikely that random variations in this variable may explain the observed patterns. In other words, variation in mitogenomic replication and repair mechanisms in bilaterians is probably also tightly associated with the variability in purifying selection pressures, which are at least partially driven by the selection for mitogenomic metabolic efficiency. For example, it has been proposed previously that elevated evolutionary rates in nematodes may be attributable to the loss of a mitochondrial replicase subunit [64]. As the entire phylum exhibits a limited locomotory capacity, this would explain why such a loss was not strongly selected against, as it would have been in a highly locomotory lineage.

Several previous studies attributed elevated evolutionary rates in parasites to host-parasite arms race [33,49,65–68]. Theoretical studies found indications that this may result in runaway evolution in parasites and hosts retreating from the arms race [69,70]. However, we find it unlikely that mitochondrial genomes are directly involved in the host-parasite arms race, as they don't encode any key genes in this aspect. Indeed, our analyses indicate that both free-living and parasitic lineages are undergoing directional selection. Flatworms were an exception, as only parasitic lineages exhibited signs of directional selection. We did find evidence that parasitic lineages exhibit relaxed purifying selection pressures compared to the free-living lineages. It is possible that relaxed purifying selection pressures allow an increased number of mutations to accumulate in parasitic organisms, which in turn provides a rich material for directional selection to work on compared to lineages evolving under strict purifying selection pressures. Intriguingly, rates of adaptive substitution are substantially higher in invertebrates than in vertebrates [50,71], which may also be a reflection of the above mechanism.

Finally, a variable that received limited scientific attention so far is the reduction of metabolic and genomic complexity in some parasites, putatively in combination with high metabolic dependence on the host [72–74]. Herein, we hypothesised that this may allow a degradation of the mitogenomic energy production efficiency, i.e. relaxed purifying selection pressures in some parasitic lineages.

Additional discussion of limitations and confounding factors

As our dataset did not conform to the assumption of independence of data (phylogenetic relatedness), for pairwise analyses, we conducted both ordinary (Tukey HSD) and phylogeny-corrected statistical tests (PGLS ANOVA). We found evidence that PGLS ANOVA may have

overestimated the statistical significance of differences. This has been observed before, and it was proposed that in such cases traditional non-phylogenetically controlled approaches might be statistically more appropriate [38], so we largely relied on the standard Tukey HSD test. In addition, multilevel regression analyses were barely affected by accounting for the phylogenetic relatedness of data.

Statistical analyses were negatively affected by the nonnormality of branch length distribution (all endoparasites were identified as outliers). We attempted to address this by log-transforming the data for multilevel regression analyses.

Some of the noise at lower taxonomic levels may putatively be attributable to the fact that we only constrained the topology at the phylum level, so lower taxonomic levels are certainly plagued by long-branch attraction artefacts [75,76]. Also, many parasitic lineages comprise free-living larval stages [24]. Most of these possess merely a rudimentary locomotory capacity, but we may have overlooked some lineages that evolve under selection for locomotory capacity during their free-living larval stages.

We treated mitogenomes as a single marker due to the (mostly) absence of recombination, unilinear inheritance, and the fact that all PCGs are involved in the same (OXPHOS) pathway, but mitochondrial genes exhibit a relatively broad variability in evolutionary rates, commonly with *cox1* being the most conserved and *atp8* the least conserved [67]. However, this should not be artefactual results, as the same genes are commonly slow-evolving (e.g. *cox1*) and fast-evolving across all bilaterian lineages (e.g. *nad6*) [10,67]. Nevertheless, to corroborate this, we also analysed individual gene trees, and compared them to the one inferred using all 12 PCGs. We found that only three fast-evolving genes exhibited low correlation to the main tree, which was caused by very low levels of homology in these genes across the Bilateria. Regardless, even the results inferred using the fast-evolving *nad2* gene support our findings (Supplementary Note 6 herein). This confirms that despite the notable intergenic variability in evolutionary rates, the effects of the two studied variables are consistent across individual genes.

As the demand for locomotion is also generally highly correlated with the mitochondrial abundance in locomotory muscles [77], it could be argued that species with reduced OXPHOS efficiency could simply compensate for this by increasing the number of mitochondria in their muscles. However, building and operating an increased number of mitochondria is a costly adaptation, so this would still affect the fitness of the individual. Therefore, mitogenomes with high energy production efficiency are more adaptive regardless of the number of mitochondria in muscle cells.

Episodic evolution may be an additional confounding factor. For example, the exceptionally long branch of parasitic flatworms is partly caused by the disproportionately long stem branch of Neodermata [27]. This implies that the transition to parasitism somewhere after the Cambrian explosion in this lineage was accompanied by a prolonged period of elevated evolutionary rates. This can affect a range of analyses. However, the fact that macroparasites exhibit longer branches across lineages indicates that there is a common underlying cause for this.

As reverse substitutions are better detected by increased taxonomic sampling, denser-sampled clades might also exhibit longer branches. To test whether this may have affected our results, we inferred the correlation between the number of species per phylum and the average root-to-tip branch length per phylum. The correlation coefficient was negative ($r = -0.23$) and nonsignificant (p-value = 0.27). Indeed, a vast majority of species belonged to Chordata and Arthropoda, most of which are free-living, and both of which exhibited some of the shortest branches in the dataset. Therefore, we can reject the hypothesis that our findings are an artefact caused by a better sampling of parasitic species.

Supplementary Methods

Datasets

The mitogenomes NC_059325, NC_059324, NC_054728, NC_053523, NC_050197, NC_046603, NC_044186, and NC_024698 were removed because the sequence was missing. We removed mitogenomes of several hybrids: NC_015838 (*Xenocypris davidi* x *Megalobrama amblycephala*), NC_028224 (*Megalobrama amblycephala* x *Megalobrama pellegrini*), NC_013995 (triploid *Megalobrama amblycephala* x *Xenocypris davidi*), NC_013994 (diploid *Megalobrama amblycephala* x *Xenocypris davidi*), and NC_028224 (*Megalobrama amblycephala* x *Megalobrama pellegrini*). Several mitogenomes nominally belonging to different species were identical, which made us suspect species misidentification artefacts. In these cases, we randomly removed one species. Identical pairs were: NC_031633 and NC_013074 (removed) (different genera), NC_047465 (removed) and NC_056102 (different genera), NC_040293 (removed) and NC_008534 (same genus), NC_024623 (removed) and NC_024645 (two hybrids with one conspecific parent), NC_020760 and NC_030175 (removed) (same genus), NC_036033 (removed) and NC_036034 (hybrids with same parent species), NC_015191 (removed) and NC_020011 (same genus), NC_026581 and NC_051547 (removed) (same genus), and NC_053682 and NC_053663 (removed) (same genus). We also removed several

fragmented mitogenomes: *Anaticola crassicornis* NC_015998, *Liposcelis_entomophila* NC_025504 and NC_025503, and *Brachionus_plicatilis* NC_010472 and NC_010484. Finally, we removed NC_012980 because a conspecific (*Carassius auratus*) mitogenome already exists in the dataset.

We relied on the default GenBank taxonomic identity, retrieved from the NCBI's taxonomy database. As this is not regularly updated, there may be some minor differences with currently accepted taxonomy. For example, in the NCBI's database, Acanthocephala and Rotifera are two stand-alone phyla, but in recent classifications, they form a phylum Syndermata [78,79]. The dataset comprised all valid phyla aside from two small ones: Loricifera (37 species in total) and Micrognathozoa (only one known species).

To assess whether the nominally bilaterian mitogenomic dataset downloaded from GenBank contained some mislabelled nonbilaterian mitogenomes, we compared root-to-tip branch lengths of all species in the dataset and inferred long-branch scores (which rely on patristic distances) [80] and "spurious species" (which rely on terminal branch lengths) using TreeSuite function in Phylosuite v1.2.3 [81]. The longest 140 branches and highest long-branch scores were all found in Platyhelminthes, and there were no outliers. Spurious species identification produced a different result. The longest terminal branch was exhibited by *Intoshia linei* (NC_060553). NCBI BLAST indicated that the closest species, albeit with low similarities of around 74%, are all Arthropoda (Insecta or Acari). This species was the only representative of the phylum Orthonectida, which caused this exceptionally long terminal branch. The second-highest-ranked spurious species was *Lingula anatina* (NC_036679). This species belongs to Brachiopoda, but it has a much longer branch and much larger mitogenome than other species from this phylum included in the dataset. BLAST resolved a large number of *Anatina* sequences as the closest hit. The third highest-ranked spurious species was *Didesmococcus koreanus* (Insecta: Coccidae (NC_057479). BLAST resolved a large number of other Coccidae sequences as the closest hits. The fourth highest-ranked spurious species was *Spirobranchus giganteus* (Annelida: Sedentaria) (NC_032055). BLAST produced somewhat "scattered" results, with species from Sedentaria, Bivalvia, and Arachnida resolved as closest hits. We also searched for stop codons in all species to assess whether any species might be using a different code than the one expected given its nominal taxonomic identity. Stop codons were found in only two genes of two different species: 29 stop codons in *nad2* of a snail species *Tritia obsoleta* (NC_007781) (29 stop codons) and 4 stop codons in *nad4L* of a turtle species *Pelomedusa subrufa* (NC_001947). This indicates that these two genes are misannotated or that there are sequencing artefacts causing frameshift mutations. Therefore, there are no indications that any nonbilaterian species were mistakenly included in the dataset.

This does not include outgroups for phylogenetic analyses, for which we selected the four most closely related phyla to Bilateria [82]: Ctenophora, Porifera, Cnidaria, Placozoa (two mitogenomes each). The eight species were: *Metridium senile* (NC_000933), *Acropora tenuis* (NC_003522), *Hoilungia* sp. (MT957399), *Polyplacotoma mediterranea* (NC_041549), *Axinella corrugata* (NC_006894), *Geodia_neptuni* (NC_006990), *Mnemiopsis leidyi* (NC_016117), *Coeloplana loyai* (LN898113).

Categorisation of life-history (LHT) strategies

All plant parasites were included in the free-living category, although some, such as plant-parasitic nematodes, may be similar to those classified as parasitic in terms of reduced locomotory capacity and confinement to a single host. Due to the high overall similarity between parasitoid and parasitic castrator strategies [83], the latter were also classified as parasitoids in most cases. Exceptions were some cestodes, which exhibit a mix of endoparasitic and parasitic castrator strategies [84]. With respect to our hypothesis (locomotory capacity, metabolic dependence on the host, and physical confinement to a single host) they are more similar to endoparasites than to parasitoids, so they were classified as endoparasites. We assigned Oestridae to parasitoids, although their classification was somewhat ambiguous with respect to our hypotheses: larvae are endoparasitic, and adults are free-living, but adults generally do not feed during their short life. Therefore, the lineage could be treated as strictly parasitic as far as diet is concerned, but not as far as locomotory capacity and confinement to a single host are concerned. A micropredator attacks more than one host (but one at a time), reducing each host's fitness by at least a small amount. Most micropredators are haematophagous (feeding on blood). They include annelids such as leeches, many insects (such as mosquitoes, tsetse flies and bed bugs), and even some vertebrates, such as lampreys and vampire bats. They often exhibit a mix of feeding strategies. For example, in many mosquitoes (Culicidae), typically both males and females feed non-parasitically. Only females need a blood meal in order to obtain the nutrients needed to produce eggs. Oxpecker *Buphagus erythrorhynchus* (Aves) is also such an example; their relationship with mammals was originally thought to be an example of mutualism, but more recent evidence suggests that oxpeckers may be facultative parasites (micropredators) [85]. Following the above definition, sheep ked *Melophagus ovinus* was classified as an ectoparasite and not as a micropredator, because it is believed to spend its entire life in the wool of a single sheep [26]. *Tinaminyssus melloi* and *Ptilonyssus chloris* (Arachnida: Mesostigmata: Rhinonyssidae) were classified as endoparasites. Nasal mites show characteristics typical of endoparasitic species: reduced shielding, reduced setation, and overall, a body type that would have

reduced mobility compared to free-living or ectoparasitic relatives [86]. *Trouessartia rubecula*, a feather mite, lives on birds' feathers, but feeds on feather oils, so we did not classify it as parasitic. The glochidium (plural glochidia) is a microscopic larval stage of some freshwater mussels, aquatic bivalve molluscs in the families Unionidae and Margaritiferidae, the river mussels and European freshwater pearl mussels. This larva form has hooks, which enable it to attach itself to fish (for example to the gills of a fish host species) for a period before it detaches and falls to the substrate and takes on the typical form of a juvenile mussel. Since a fish is active and free-swimming, this process helps distribute the mussel species to areas that it could not reach otherwise. Despite its temporary attachment to the host, we did not classify any of these species as parasitic. We failed to find sufficiently precise life-history information for *Goniophyto honshuensis, Miltogramma oestracea* (Sarcophagidae) and *Canthesancus helluo* (Reduviidae). As larvae of some Sarcophagidae are internal parasites of other insects such as Orthoptera, and some Reduviidae are blood-sucking ectoparasites, we did not assign an LHT category to these three species, and we removed them from the dataset used for statistical analyses. We also removed five Strongyloididae (Nematoda) species from this dataset because they alternate between free-living and parasitic stages. Our initial analyses indicated that they exhibit almost the same branch lengths as strictly endoparasitic nematodes.

Categorisation of locomotory capacity (LOC)

Chaetognaths swim in short bursts using a dorso-ventral undulating body motion, where their tail fin assists with propulsion and the body fins with stabilization and steering, so we classified them into the Intermediate category. Annelida were difficult to classify. Sipuncula merely have a rudimentary LOC, so they were classified as Low. Crassiclitellata (earthworms), Hirudinida (leeches) and Eunicida are capable of more than rudimentary locomotion, so we classified them as Intermediate. Sabellida are sedentary, so we classified them as Low. Terebellida and Urechidae appear to possess merely a rudimentary LOC, so we placed them into the Low category. Magelonidae are burrowing worms with limited mobility, so we classified them as Low. Some Nemertea species may possess more than a basic LOC, but due to limited evidence, we classified them all as Low. Phoronida, Priapulida, Entoprocta, and Hemichordata possess merely a rudimentary locomotory capacity, so all were classified as Low. Onychophora can move at up to 5 cm per second, so we classified them as Intermediate.

Phylogenetic analyses

To make sure that our results were not affected by phylogenetic reconstruction artefacts (variable branch length depending on the method used), the phylogeny of Bilateria was inferred using IQ-TREE v2.2.0.7.mix [87] and FastTree v2.1.10 [88] programs, in combination with several different datasets and strategies. FastTree analyses were conducted using the AAs dataset, comprising amino acid sequences of 12 mitochondrial protein-coding genes (*atp8* was removed because it is missing from multiple lineages) and two different evolutionary models: LG+G (inferred as the optimal model using the AICc criterion) and JTT+G (inferred as the optimal model using the BIC criterion). IQ-TREE was tested in combination with three different datasets, and multiple strategies. The datasets were: 1) The AAs dataset; 2) The AAs dataset with fast-evolving, difficult to align, genes removed: *nad2*, *nad4L* and *nad6* (AAs_9genes); 3) The nucleotide dataset, comprising only 1st and 2nd codon sites of protein-coding genes (NUC12). All datasets comprised 10,911 mitogenomes along with 8 non-bilaterian Animalia outgroup species. All sequences were aligned using MAFFT [89], poorly aligned sections were removed from alignments using trimAI [90] (AAs datasets) and Gblocks [91] (NUC12 dataset), and genes were then concatenated by PhyloSuite. For the AAs dataset, the optimal model selection was performed using ModelFinder (available in IQ-TREE v2.2.0.7.mix) [92]. PhyloSuite was used to split nucleotide sequences of genes by the codon position and remove the third codon position. We applied a number of IQ-TREE strategies to assess the stability of branch lengths. Node support was inferred using the approximate Bayes test [93] in all analyses. Given the computational limitations of our (very large) dataset, first we tested whether the "-fast" IQ-TREE runs produce congruent results with computationally much more demanding standard runs using both datasets. For the AAs dataset, we used the mtZOA+G4 model, where mtZOA is a model specifically designed for mitochondrial data [94], and the G4 parameter was identified as optimal by ModelFinder [92]. This parameter refers to a model of rate heterogeneity that uses a discrete gamma distribution with four rate categories to model the variation in evolutionary rates among different sites. The "-fast" mode and the standard mode produced trees with root-to-tip branch lengths exhibiting a 98.6% correlation. For the NUC12 dataset, we used the GTR+G+I model, following the evidence that the use of this, the most parameter-rich evolutionary model, allows skipping the evolutionary model selection step in phylogenetic analyses without producing any detrimental effects on the accuracy of topology [95]. The "-fast" mode and the standard mode produced NUC12 trees with root-to-tip branch lengths exhibiting 99.8% correlation. As this step provided relatively solid evidence that the use of a computationally less demanding "-fast" model has a minimal effect on branch lengths, we relied on this algorithm to conduct two more runs of the AAs dataset. We decided to test the AAs dataset further because AAs are generally more conserved than nucleotides, which makes them easier to

align in datasets comprising very deep evolutionary splits. In addition, AAs and NUC12 datasets produced highly congruent trees, with branch length correlations of 96.2-97.4%, which indicates that the choice of dataset would have minimal impacts on our analyses and conclusions. FastTree topologies exhibited 90 to 97% correlation with the IQ-TREE topologies, which also indicates that results would be mostly congruent regardless of which topology was used to conduct downstream analyses. As there are multiple lines of evidence that amino acid alignments in combination with models designed for compositional heterogeneity produce better results on complex datasets [96–98], we further used the protein mixture model C50 [99] implemented in IQ-TREE (mtZOA+C50+F+G). It is a variant of the CAT model, designed to account for substitutional heterogeneity [100], and it assumes a Poisson AA replacement and a Gamma rate heterogeneity among sites. Importantly, this model is expected to produce more accurate branch length estimates in deep phylogenies due to improved modelling of mutational convergences and reversions [98]. Finally, among the individual gene trees, *nad2*, *nad4L*, and *nad6* exhibited a low correlation with the mitogenomic tree. These are fast-evolving genes, so they exhibited a limited homology across the bilaterian dataset. We tested their impact by removing them from the dataset (AAs_9genes) and reconducting the mtZOA+G4+F+C50 (referred to as C50 in the paper) analysis. The two trees produced branch lengths with a 99% correlation, indicating that the inclusion of these three genes did not affect the overall results. Therefore, the AAs 12PCGs C50 tree was used for all analyses.

Datasets used for selection tests

As the phylogenetic distribution of endoparasitic lineages employing the Table 5 code spanned three phyla, we tested a dataset comprising all endoparasitic and a selection of free-living species across nine phyla (N = 220). Ectoparasitic lineages in the Table 5 dataset were limited to Arthropoda, so we selected a subdataset in the same way, with free-living lineages sampled only from Arthropoda (N = 178). There were too few (6) endoparasitic species in the Arthropoda dataset, and they were included in the Table 5 dataset, so we did not test them separately. However, we did test the Nematoda dataset (Table 5) separately, as this phylum comprised a mix of endoparasitic and free-living species (N = 174). The only parasitic lineages employing other genetic codes (Table 9) were limited to the phylum Platyhelminthes. As the number of free-living species in this dataset was low (10), to improve the statistical power of analyses, we used a dataset from our recent study [27], as it comprised all available flatworm mitogenomes, including 30 free-living species.

Supplementary References

1. Zou, H. *et al.* Inverted base composition skews and discontinuous mitochondrial genome architecture evolution in the Enoplea (Nematoda). *BMC Genomics* **23**, 376 (2022).
2. Zou, H. *et al.* Evolutionary rates of mitochondrial sequences and gene orders in Spirurina (Nematoda) are episodic but synchronised. *Water Biology and Security* **1**, 100033 (2022).
3. Zou, H. *et al.* The complete mitochondrial genome of parasitic nematode Camallanus cotti: extreme discontinuity in the rate of mitogenomic architecture evolution within the Chromadorea class. *BMC Genomics* **18**, 840 (2017).
4. Chen, F. *et al.* Sequencing of the Complete Mitochondrial Genome of Pingus sinensis (Spirurina: Quimperiidae): Gene Arrangements and Phylogenetic Implications. *Genes* **12**, 1772 (2021).
5. Zou, H. *et al.* The complete mitochondrial genome of Cymothoa indica has a highly rearranged gene order and clusters at the very base of the Isopoda clade. *PLOS ONE* **13**, e0203089 (2018).
6. Zou, H. *et al.* Architectural instability, inverted skews and mitochondrial phylogenomics of Isopoda: outgroup choice affects the long-branch attraction artefacts. *Royal Society Open Science* **7**, 191887 (2020).
7. Hua, C. J. *et al.* Basal position of two new complete mitochondrial genomes of parasitic Cymothoida (Crustacea: Isopoda) challenges the monophyly of the suborder and phylogeny of the entire order. *Parasites & Vectors* **11**, 628 (2018).
8. Zhang, D. *et al.* Mitochondrial genomes and 28S rDNA contradict the proposed obsoletion of the order Tetraonchidea (Platyhelminthes: Monogenea). *International Journal of Biological Macromolecules* **143**, 891–901 (2020).
9. Zhang, D. *et al.* Evidence for Adaptive Selection in the Mitogenome of a Mesoparasitic Monogenean Flatworm Enterogyrus malmbergi. *Genes* **10**, 863 (2019).
10. Zhang, D. *et al.* Mitochondrial genomes of two diplectanids (Platyhelminthes: Monogenea) expose paraphyly of the order Dactylogyridea and extensive tRNA gene rearrangements. *Parasites & Vectors* **11**, 601 (2018).
11. Zhang, D. *et al.* Three new Diplozoidae mitogenomes expose unusual compositional biases within the Monogenea class: implications for phylogenetic studies. *BMC Evolutionary Biology* **18**, 133 (2018).
12. Zhang, D. *et al.* Sequencing of the complete mitochondrial genome of a fish-parasitic flatworm Paratetraonchoides inermis (Platyhelminthes: Monogenea): tRNA gene arrangement reshuffling and implications for phylogeny. *Parasites & Vectors* **10**, 462 (2017).
13. Zhang, D. *et al.* Sequencing, characterization and phylogenomics of the complete mitochondrial genome of Dactylogyrus lamellatus (Monogenea: Dactylogyridae). *Journal of Helminthology* 1–12 (2017) doi:10.1017/S0022149X17000578.
14. Zhang, D. *et al.* Mitochondrial Genomes of Two Thaparocleidus Species (Platyhelminthes: Monogenea) Reveal the First rRNA Gene Rearrangement among the Neodermata. *International Journal of Molecular Sciences* **20**, 4214 (2019).
15. Zhang, D. *et al.* Homoplasy or plesiomorphy? Reconstruction of the evolutionary history of mitochondrial gene order rearrangements in the subphylum Neodermata. *International Journal for Parasitology* **49**, 819–829 (2019).
16. Li, W. X. *et al.* Characterization and phylogenomics of the complete mitochondrial genome of the polyzoic cestode Gangesia oligonchis (Platyhelminthes: Onchoproteocephalidea). *Journal of Helminthology* **94**, e58 (2020).
17. Li, W. X. *et al.* The complete mitochondrial DNA of three monozoic tapeworms in the Caryophyllidea: a mitogenomic perspective on the phylogeny of eucestodes. *Parasites & Vectors* **10**, 314 (2017).
18. Li, W. X. *et al.* Comparative mitogenomics supports synonymy of the genera Ligula and Digramma (Cestoda: Diphyllobothriidae). *Parasites & Vectors* **11**, 324 (2018).

19. Zou, H. *et al.* The complete mitochondrial genome of Gyrodactylus gurleyi (Platyhelminthes: Monogenea). *Mitochondrial DNA Part B* **1**, 383–385 (2016).
20. Zhang, D. *et al.* The complete mitochondrial genome of Gyrodactylus kobayashii (Platyhelminthes: Monogenea). *Mitochondrial DNA Part B* **1**, 146–147 (2016).
21. Meade, A. & Pagel, M. Ancestral State Reconstruction Using BayesTraits. in *Environmental Microbial Evolution: Methods and Protocols* (ed. Luo, H.) 255–266 (Springer US, 2022). doi:10.1007/978-1-0716-2691-7_12.
22. Blaxter, M. L. *et al.* A molecular evolutionary framework for the phylum Nematoda. *Nature* **392**, 71–75 (1998).
23. Blaxter, M. & Koutsovoulos, G. The evolution of parasitism in Nematoda. *Parasitology* **142**, S26–S39 (2015).
24. Weinstein, S. B. & Kuris, A. M. Independent origins of parasitism in Animalia. *Biology Letters* **12**, 20160324 (2016).
25. Jakovlić, I. *et al.* Slow crabs - fast genomes: locomotory capacity predicts skew magnitude in crustacean mitogenomes. *Molecular Ecology* **30**, 5488–5502 (2021).
26. Small, R. W. A review of Melophagus ovinus (L.), the sheep ked. *Veterinary Parasitology* **130**, 141–155 (2005).
27. Jakovlić, I. *et al.* Drivers of interlineage variability in mitogenomic evolutionary rates in flatworms (Platyhelminthes) are multifactorial. 2022.09.11.507443 Preprint at https://doi.org/10.1101/2022.09.11.507443 (2022).
28. Martin, A. P. & Palumbi, S. R. Body size, metabolic rate, generation time, and the molecular clock. *PNAS* **90**, 4087–4091 (1993).
29. Gillooly, J. F., Allen, A. P., West, G. B. & Brown, J. H. The rate of DNA evolution: Effects of body size and temperature on the molecular clock. *PNAS* **102**, 140–145 (2005).
30. Nabholz, B., Lanfear, R. & Fuchs, J. Body mass-corrected molecular rate for bird mitochondrial DNA. *Molecular Ecology* **25**, 4438–4449 (2016).
31. Thomas, J. A., Welch, J. J., Woolfit, M. & Bromham, L. There is no universal molecular clock for invertebrates, but rate variation does not scale with body size. *PNAS* **103**, 7366–7371 (2006).
32. Lanfear, R., Thomas, J. A., Welch, J. J., Brey, T. & Bromham, L. Metabolic rate does not calibrate the molecular clock. *PNAS* **104**, 15388–15393 (2007).
33. Thomas, J. A., Welch, J. J., Lanfear, R. & Bromham, L. A Generation Time Effect on the Rate of Molecular Evolution in Invertebrates. *Mol Biol Evol* **27**, 1173–1180 (2010).
34. Nabholz, B., Glémin, S. & Galtier, N. Strong Variations of Mitochondrial Mutation Rate across Mammals—the Longevity Hypothesis. *Mol Biol Evol* **25**, 120–130 (2008).
35. Galtier, N., Jobson, R. W., Nabholz, B., Glémin, S. & Blier, P. U. Mitochondrial whims: metabolic rate, longevity and the rate of molecular evolution. *Biology Letters* **5**, 413–416 (2009).
36. Welch, J. J., Bininda-Emonds, O. R. & Bromham, L. Correlates of substitution rate variation in mammalian protein-coding sequences. *BMC Evolutionary Biology* **8**, 53 (2008).
37. Moosmann, B. & Behl, C. Mitochondrially encoded cysteine predicts animal lifespan. *Aging Cell* **7**, 32–46 (2008).
38. Huang, D., Meier, R., Todd, P. A. & Chou, L. M. Slow Mitochondrial COI Sequence Evolution at the Base of the Metazoan Tree and Its Implications for DNA Barcoding. *J Mol Evol* **66**, 167–174 (2008).
39. Rand, D. M. Thermal habit, metabolic rate and the evolution of mitochondrial DNA. *Trends in Ecology & Evolution* **9**, 125–131 (1994).
40. Wang, Y. & Hekimi, S. Mitochondrial dysfunction and longevity in animals: Untangling the knot. *Science* **350**, 1204–1207 (2015).
41. Allio, R., Donega, S., Galtier, N. & Nabholz, B. Large variation in the ratio of mitochondrial to nuclear mutation rate across animals: implications for genetic diversity and the use of mitochondrial DNA as a molecular marker. *Mol Biol Evol* **34**, 2762–2772 (2017).

42. Saclier, N. *et al.* Life history traits impact the nuclear rate of substitution but not the mitochondrial rate in isopods. *Molecular Biology and Evolution* **35**, 2900–2912 (2018).
43. Galvani, A. P., Coleman, R. M. & Ferguson, N. M. The maintenance of sex in parasites. *Proceedings of the Royal Society of London. Series B: Biological Sciences* **270**, 19–28 (2003).
44. Jaenike, J. Mycophagous Drosophila and Their Nematode Parasites. *The American Naturalist* (1992) doi:10.1086/285365.
45. Lynch, M. The Evolution of Cladoceran Life Histories. *The Quarterly Review of Biology* **55**, 23–42 (1980).
46. Lynch, M., Koskella, B. & Schaack, S. Mutation pressure and the evolution of organelle genomic architecture. *Science* **311**, 1727–1730 (2006).
47. Lanfear, R., Ho, S. Y. W., Love, D. & Bromham, L. Mutation rate is linked to diversification in birds. *PNAS* **107**, 20423–20428 (2010).
48. Hardouin, E. a & Tautz, D. Increased mitochondrial mutation frequency after an island colonization: positive selection or accumulation of slightly deleterious mutations? *Biology Letters* **9**, 20121123–20121123 (2013).
49. James, J. E., Piganeau, G. & Eyre-Walker, A. The rate of adaptive evolution in animal mitochondria. *Mol Ecol* **25**, 67–78 (2016).
50. Bazin, E., Glémin, S. & Galtier, N. Population Size Does Not Influence Mitochondrial Genetic Diversity in Animals. *Science* **312**, 570–572 (2006).
51. Lynch, M. & Conery, J. S. The Origins of Genome Complexity. *Science* **302**, 1401–1404 (2003).
52. Palstra, F. P. & Ruzzante, D. E. Genetic estimates of contemporary effective population size: what can they tell us about the importance of genetic stochasticity for wild population persistence? *Molecular Ecology* **17**, 3428–3447 (2008).
53. Nabholz, B., Glémin, S. & Galtier, N. The erratic mitochondrial clock: variations of mutation rate, not population size, affect mtDNA diversity across birds and mammals. *BMC Evol Biol* **9**, 54 (2009).
54. Melvin, R. G. & Ballard, J. W. O. Cellular and population level processes influence the rate, accumulation and observed frequency of inherited and somatic mtDNA mutations. *Mutagenesis* **32**, 323–334 (2017).
55. Lajbner, Z., Pnini, R., Camus, M. F., Miller, J. & Dowling, D. K. Experimental evidence that thermal selection shapes mitochondrial genome evolution. *Scientific Reports* **8**, 9500 (2018).
56. Thomas, W. K. & Beckenbach, A. T. Variation in salmonid mitochondrial DNA: Evolutioinary constraints and mechanisms of substitution. *J Mol Evol* **29**, 233–245 (1989).
57. Lagisz, M., Poulin, R. & Nakagawa, S. You are where you live: parasitic nematode mitochondrial genome size is associated with the thermal environment generated by hosts. *Journal of Evolutionary Biology* **26**, 683–690 (2013).
58. Martin, A. P. Metabolic rate and directional nucleotide substitution in animal mitochondrial DNA. *Molecular Biology and Evolution* **12**, 1124–1131 (1995).
59. Faith, J. J. & Pollock, D. D. Likelihood Analysis of Asymmetrical Mutation Bias Gradients in Vertebrate Mitochondrial Genomes. *Genetics* **165**, 735–745 (2003).
60. Touchon, M., Arneodo, A., d'Aubenton-Carafa, Y. & Thermes, C. Transcription-coupled and splicing-coupled strand asymmetries in eukaryotic genomes. *Nucleic Acids Res* **32**, 4969–4978 (2004).
61. Britten, R. J. Rates of DNA sequence evolution differ between taxonomic groups. *Science* **231**, 1393–1398 (1986).
62. Christensen, A. C. Genes and Junk in Plant Mitochondria—Repair Mechanisms and Selection. *Genome Biol Evol* **6**, 1448–1453 (2014).
63. Lewis, S. C. *et al.* A Rolling Circle Replication Mechanism Produces Multimeric Lariats of Mitochondrial DNA in Caenorhabditis elegans. *PLOS Genetics* **11**, e1004985 (2015).
64. Oliveira, M. T., Haukka, J. & Kaguni, L. S. Evolution of the Metazoan Mitochondrial Replicase. *Genome Biol Evol* **7**, 943–959 (2015).

65. Dowton, M. & Austin, A. D. Increased genetic diversity in mitochondrial genes is correlated with the evolution of parasitism in the Hymenoptera. *J Mol Evol* **41**, 958–965 (1995).
66. Huyse, T., Poulin, R. & Théron, A. Speciation in parasites: a population genetics approach. *Trends in Parasitology* **21**, 469–475 (2005).
67. da Fonseca, R. R., Johnson, W. E., O'Brien, S. J., Ramos, M. J. & Antunes, A. The adaptive evolution of the mammalian mitochondrial genome. *BMC Genomics* **9**, 119 (2008).
68. Oliveira, D. C. S. G., Raychoudhury, R., Lavrov, D. V. & Werren, J. H. Rapidly Evolving Mitochondrial Genome and Directional Selection in Mitochondrial Genes in the Parasitic Wasp Nasonia (Hymenoptera: Pteromalidae). *Mol Biol Evol* **25**, 2167–2180 (2008).
69. Haraguchi, Y. & Sasaki, A. Host–Parasite Arms Race in Mutation Modifications: Indefinite Escalation Despite a Heavy Load? *Journal of Theoretical Biology* **183**, 121–137 (1996).
70. Dawkins, R. & Krebs, J. R. Arms races between and within species. *Proceedings of the Royal Society of London. Series B, Containing papers of a Biological character. Royal Society (Great Britain)* **205**, 489–511 (1979).
71. Meiklejohn, C. D., Montooth, K. L. & Rand, D. M. Positive and negative selection on the mitochondrial genome. *Trends in Genetics* **23**, 259–263 (2007).
72. Keeling, P. J. *et al.* The Reduced Genome of the Parasitic Microsporidian Enterocytozoon bieneusi Lacks Genes for Core Carbon Metabolism. *Genome Biol Evol* **2**, 304–309 (2010).
73. Poulin, R. & Randhawa, H. S. Evolution of parasitism along convergent lines: from ecology to genomics. *Parasitology* **142**, S6–S15 (2015).
74. Slyusarev, G. S., Starunov, V. V., Bondarenko, A. S., Zorina, N. A. & Bondarenko, N. I. Extreme Genome and Nervous System Streamlining in the Invertebrate Parasite Intoshia variabili. *Current Biology* **30**, 1292-1298.e3 (2020).
75. Rota-Stabelli, O. *et al.* Ecdysozoan Mitogenomics: Evidence for a Common Origin of the Legged Invertebrates, the Panarthropoda. *Genome Biology and Evolution* **2**, 425–440 (2010).
76. Jakovlić, I. *et al.* Evolutionary History of Inversions in Directional Mutational Pressures in Crustacean Mitochondrial Genomes: Implications for Evolutionary Studies. *Molecular Phylogenetics and Evolution* **164**, 107288 (2021).
77. Childress, J. J. Are there physiological and biochemical adaptations of metabolism in deep-sea animals? *Trends in Ecology & Evolution* **10**, 30–36 (1995).
78. Sielaff, M. *et al.* Phylogeny of Syndermata (syn. Rotifera): Mitochondrial gene order verifies epizoic Seisonidea as sister to endoparasitic Acanthocephala within monophyletic Hemirotifera. *Molecular Phylogenetics and Evolution* **96**, 79–92 (2016).
79. Monks, S. Phylogeny of the Acanthocephala based on morphological characters. *Syst Parasitol* **48**, 81–115 (2001).
80. Struck, T. H. TreSpEx—Detection of Misleading Signal in Phylogenetic Reconstructions Based on Tree Information. *Evol Bioinform Online* **10**, 51–67 (2014).
81. Xiang, C. *et al.* Using PhyloSuite for molecular phylogeny and tree-based analyses. *iMeta* e87 (2023) doi:10.1002/imt2.87.
82. Laumer, C. E. *et al.* Revisiting metazoan phylogeny with genomic sampling of all phyla. *Proceedings of the Royal Society B: Biological Sciences* **286**, 20190831 (2019).
83. Kuris, A. M. Trophic Interactions: Similarity of Parasitic Castrators to Parasitoids. *The Quarterly Review of Biology* **49**, 129–148 (1974).
84. Arnott, S. A., Barber, I. & Huntingford, F. A. Parasite–associated growth enhancement in a fish–cestode system. *Proceedings of the Royal Society of London. Series B: Biological Sciences* **267**, 657–663 (2000).
85. Plantan, T., Howitt, M., Kotzé, A. & Gaines, M. Feeding preferences of the red-billed oxpecker, Buphagus erythrorhynchus: a parasitic mutualist? *African Journal of Ecology* **51**, 325–336 (2013).
86. Hilario-Pérez, A. D. & Dowling, A. P. G. Nasal mites from specimens of the brown-headed cowbird (Icteridae: Molothrus ater) from Texas and Arkansas, U.S.A. *Acarologia* **58**, 296–301 (2018).

87. Minh, B. Q. *et al.* IQ-TREE 2: New Models and Efficient Methods for Phylogenetic Inference in the Genomic Era. *Molecular Biology and Evolution* **37**, 1530–1534 (2020).
88. Price, M. N., Dehal, P. S. & Arkin, A. P. FastTree 2 – Approximately Maximum-Likelihood Trees for Large Alignments. *PLOS ONE* **5**, e9490 (2010).
89. Katoh, K. & Standley, D. M. MAFFT multiple sequence alignment software version 7: Improvements in performance and usability. *Molecular Biology and Evolution* **30**, 772–780 (2013).
90. Capella-Gutiérrez, S., Silla-Martínez, J. M. & Gabaldón, T. trimAl: a tool for automated alignment trimming in large-scale phylogenetic analyses. *Bioinformatics* **25**, 1972–1973 (2009).
91. Castresana, J. Selection of Conserved Blocks from Multiple Alignments for Their Use in Phylogenetic Analysis. *Molecular Biology and Evolution* **17**, 540–552 (2000).
92. Kalyaanamoorthy, S., Minh, B. Q., Wong, T. K. F., Von Haeseler, A. & Jermiin, L. S. ModelFinder: Fast model selection for accurate phylogenetic estimates. *Nature Methods* **14**, 587–589 (2017).
93. Anisimova, M., Gil, M., Dufayard, J.-F., Dessimoz, C. & Gascuel, O. Survey of Branch Support Methods Demonstrates Accuracy, Power, and Robustness of Fast Likelihood-based Approximation Schemes. *Systematic Biology* **60**, 685–699 (2011).
94. Rota-Stabelli, O., Yang, Z. & Telford, M. J. MtZoa: A general mitochondrial amino acid substitutions model for animal evolutionary studies. *Molecular Phylogenetics and Evolution* **52**, 268–272 (2009).
95. Abadi, S., Azouri, D., Pupko, T. & Mayrose, I. Model selection may not be a mandatory step for phylogeny reconstruction. *Nat Commun* **10**, 1–11 (2019).
96. Zhang, D. *et al.* Mitochondrial Architecture Rearrangements Produce Asymmetrical Nonadaptive Mutational Pressures That Subvert the Phylogenetic Reconstruction in Isopoda. *Genome Biology and Evolution* **11**, 1797–1812 (2019).
97. Rodríguez-Ezpeleta, N. *et al.* Detecting and Overcoming Systematic Errors in Genome-Scale Phylogenies. *Systematic Biology* **56**, 389–399 (2007).
98. Lartillot, N., Brinkmann, H. & Philippe, H. Suppression of long-branch attraction artefacts in the animal phylogeny using a site-heterogeneous model. *BMC Evolutionary Biology* **7**, S4 (2007).
99. Si Quang, L., Gascuel, O. & Lartillot, N. Empirical profile mixture models for phylogenetic reconstruction. *Bioinformatics* **24**, 2317–2323 (2008).
100. Lartillot, N. & Philippe, H. A Bayesian Mixture Model for Across-Site Heterogeneities in the Amino-Acid Replacement Process. *Molecular Biology and Evolution* **21**, 1095–1109 (2004).
