# Supplementary material for: Mitogenomic evolutionary rates in bilateria are influenced by parasitic lifestyle and locomotory capacity
Source: Nat Commun. 2023 Oct 9;14:6307. doi: 10.1038/s41467-023-42095-8 (PMC10562372; doi:10.1038/s41467-023-42095-8)
Supplement: Supplementary file 2 — Reporting Summary [file 41467_2023_42095_MOESM2_ESM.pdf]

nature portfolio

Corresponding author(s): Dong Zhang

Last updated by author(s): May 19, 2023

Reporting Summary

Nature Portfolio wishes to improve the reproducibility of the work that we publish. This form provides structure for consistency and transparency in reporting. For further information on Nature Portfolio policies, see our Editorial Policies and the Editorial Policy Checklist.

Statistics

For all statistical analyses, confirm that the following items are present in the figure legend, table legend, main text, or Methods section.

n/a	Confirmed	
☐	☒	The exact sample size (*n*) for each experimental group/condition, given as a discrete number and unit of measurement
☒	☐	A statement on whether measurements were taken from distinct samples or whether the same sample was measured repeatedly
☐	☒	The statistical test(s) used AND whether they are one- or two-sided<br>*Only common tests should be described solely by name; describe more complex techniques in the Methods section.*
☐	☒	A description of all covariates tested
☐	☒	A description of any assumptions or corrections, such as tests of normality and adjustment for multiple comparisons
☐	☒	A full description of the statistical parameters including central tendency (e.g. means) or other basic estimates (e.g. regression coefficient) AND variation (e.g. standard deviation) or associated estimates of uncertainty (e.g. confidence intervals)
☐	☒	For null hypothesis testing, the test statistic (e.g. *F*, *t*, *r*) with confidence intervals, effect sizes, degrees of freedom and *P* value noted<br>*Give P values as exact values whenever suitable.*
☒	☐	For Bayesian analysis, information on the choice of priors and Markov chain Monte Carlo settings
☒	☐	For hierarchical and complex designs, identification of the appropriate level for tests and full reporting of outcomes
☐	☒	Estimates of effect sizes (e.g. Cohen's *d*, Pearson's *r*), indicating how they were calculated

Our web collection on statistics for biologists contains articles on many of the points above.

Software and code

Policy information about availability of computer code

Data collection	PhyloSuite was used to retrieve, standardise and extract the mitogenomic data, resolve duplicated genes, and generate comparative tables for the dataset.
Data analysis	Phylosuite v1.2.3 was used for all comparative analyses. All sequences were aligned using MAFFT v7.475, alignments trimmed by trimAl v1.2rev59, and concatenated by PhyloSuite. For the AAs dataset, the optimal model selection was performed using ModelFinder (available in IQ-TREE v2.2.0.7.mix). PhyloSuite was used to split nucleotide sequences of genes by the codon position and remove the third codon position. Phylogenetic analyses were conducted using IQ-TREE v2.2.0.7.mix and Fasttree v2.1.10. Multilevel regression analyses were conducted using the lmekin function in coxme v2.2.16 and brms v2.16.1. PGLS ANOVA tests were conducted using the nlme package v3.1.152. For pairwise comparisons of branch lengths between different groups, we conducted Tukey HSD tests using the R package agricolae (v1.3.5). The R2 value of lmekin models was calculated using the "r.squaredLR" method available in the MuMIn package v1.46.0 in R. Bayesian R2 was inferred using the bayes_r2 function of brms package in R. The AIC value of each lmekin model was calculated using the "AIC" method in R. The LRT test of lmekin models was conducted using the $\chi 2$ test following this tutorial: https://aeolister.wordpress.com/2016/07/07/likelihood-ratio-test-for-lmekin/ (version: 7/7/2016; last accessed 30/3/2023). Branch length outliers in the dataset were identified using the "boxplot" function in R. The Maximum Likelihood method in BayesTraits v4.0.1 was used to infer the ancestral states of traits. Selection pressure patterns were studied using two tools from the HYPHY suite v2.5.42: RELAX and BUSTED. The code written for filtering the mitogenome data was subsequently incorporated into PhyloSuite v. 1.2.3, available from https://github.com/dongzhang0725/PhyloSuite or https://pypi.org/project/PhyloSuite/. The remaining code written for this study was deposited in Zenodo: https://doi.org/10.5281/zenodo.7940125.

For manuscripts utilizing custom algorithms or software that are central to the research but not yet described in published literature, software must be made available to editors and reviewers. We strongly encourage code deposition in a community repository (e.g. GitHub). See the Nature Portfolio guidelines for submitting code & software for further information.

Data

Policy information about availability of data

All manuscripts must include a data availability statement. This statement should provide the following information, where applicable:

- Accession codes, unique identifiers, or web links for publicly available datasets
- A description of any restrictions on data availability
- For clinical datasets or third party data, please ensure that the statement adheres to our policy

All data used in this study were retrieved from the NCBI's GenBank RefSeq database (https://www.ncbi.nlm.nih.gov/refseq/). Source data are provided with this paper.

Human research participants

Policy information about studies involving human research participants and Sex and Gender in Research.

Reporting on sex and gender	N/A
Population characteristics	N/A
Recruitment	N/A
Ethics oversight	N/A

Note that full information on the approval of the study protocol must also be provided in the manuscript.

Field-specific reporting

Please select the one below that is the best fit for your research. If you are not sure, read the appropriate sections before making your selection.

☐ Life sciences ☐ Behavioural & social sciences ☒ Ecological, evolutionary & environmental sciences

For a reference copy of the document with all sections, see nature.com/documents/nr-reporting-summary-flat.pdf

Ecological, evolutionary & environmental sciences study design

All studies must disclose on these points even when the disclosure is negative.

Study description

We extracted 12 protein-coding genes from 10,914 mitogenomes of bilaterian animals, built a phylum-level topology-constrained phylogenetic tree using this dataset, and extracted branch lengths. The dataset was classified according to the life history into five categories: endoparasites (EndoP), ectoparasites (EctoP), parasitoids, micropredators (MP), and free-living (F). We further divided the dataset into three locomotory capacity categories. 1. High (H), comprising all species expected to rely on locomotion for pursuit and evasion of prey/predators. 2. Low (L), comprising all species that have merely a rudimentary locomotory capacity (i.e. not expected to rely on locomotion for pursuit and evasion of prey/predators). 3. Because the distinction between the high and low locomotory capacity species is blurry in many cases, we designed a third category, Intermediate LC (I), with the aim to mop up the noise produced by these difficult-to-classify taxa, and make sure that the High and Low categories do not overlap. This category comprises species that would be expected to possess more than a rudimentary locomotory capacity, but also rely on strategies other than locomotion to evade/pursue predators/prey. We organised the dataset in a hierarchical manner, by further subdividing it according to major taxonomic categories: phylum, class and order.

First we conducted pairwise comparisons of branch lengths between different groups using Tukey HSD tests and PGLS ANOVA. To assess the relative impacts of different variables on branch length, we used two multilevel regression algorithms designed to account for the phylogenetic relatedness of data: linear fixed-effect models accounting for kinship implemented in the lmekin function in coxme, and phylogenetic multilevel Bayesian models implemented in brms. For both analyses, we used a matrix of phylogenetic distances extracted from the phylogenetic tree. We log-transformed the branch length data to reduce the nonnormality of distribution. For these analyses, branch length was the dependent variable, and life history and locomotory capacity categorisations were independent variable. We also conducted analyses after removing the outliers from the dataset. Finally, we conducted selection pressure analyses.

As parasitism and locomotory capacity are partially overlapping variables, we attempted to discern their impacts using further subsets of data. To reduce the effect of locomotory capacity variability, we focused only on the Low locomotory capacity category and divided it along the life history lines. To remove the effect of parasitism on the locomotory capacity classification, we conducted analyses using only the free-living species.

Research sample

The objective of the study was to research the evolution of mitochondrial genome in bilaterian animals. Nonbilaterians possess highly divergent mitogneomes, which would have made comparative analyses difficult to conduct, and comprise only about 2% of all available animal mitogenomes, so we opted to study only bilaterian animals. The dataset was retrieved from NCBI's GenBank. The dataset for phylogenetic analysis comprised 10,914 bilaterian mitogenomes (+ 8 nonbilaterian species as outgroups), or about 2% of all recognised bilaterian species according to ITIS (521,682). The dataset for statistical analyses comprised 11,906 mitogenomes,

	because life history classification was difficult for 8 species.
Sampling strategy	When we accessed the data (10th March 2022), there were 11,284 animal and 11,017 bilaterian mitogenomes available in the RefSeq database. What is commonly recognised as the 'standard' metazoan (animal) mitogenome is a circular molecule ≈15 Kbp in size that contains 37 genes: 13 protein-coding genes (PCGs), 2 rRNA genes and 22 tRNA genes, but there are major deviations from this architecture in some lineages. While some deviations from this canon have been observed in isolated bilaterian lineages, almost all of the major discrepancies in the protein-coding gene content map to the non-bilaterian metazoans. As this would complicate some comparative analyses, and as non-bilaterian mitogenomes represented only about 2% of the total available Animalia dataset, in this study we focused only on Bilateria.
Data collection	Life history and locomotory capacity data were retrieved from a broad range of internet sources, and recorded, by Ivan Jakovlić, Chuan-Yu Xiang, and Dong Zhang.
Timing and spatial scale	Data were collected from GenBank (global) between March and May 2022. The data were not associated with spatial scale.
Data exclusions	We removed all unannotated mitogenomes, most hybrids between species, identical mitogenomes (we suspected species misidentification in these cases), and three species which we could not classify in terms of life history. From statistical analyses we excluded 5 Strongyloididae (Nematoda) species whose life cycle alternates between free-living and parasitic generations.
Reproducibility	We inferred multiple phylogenetic trees using different datasets and methods, and confirmed that all produced branch lengths with correlation over 90%. We also conducted all statistical analyses multiple times, tested the impact of removal of outliers, and confirmed that single-gene trees produce similar results. We also provided raw data and codes, allowing other scientists to reproduce our data.
Randomization	We subdivided the dataset according to major taxonomic categories, phylum, class and order, and conducted all statistical analyses on these subsets of data to ensure that overall patterns are upheld at lower taxonomic levels. As parasitism and locomotory capacity (LC) are partially overlapping variables (most but not all parasitic lineages exhibit low LC), we attempted to discern their impacts using subsets of data. To reduce the effect of locomotory capacity variability, we focused only on the Low LC category. To remove the effect of parasitism, we conducted analyses using only the free-living species. We further focused on the ectoparasitic dataset and compared lineages according to the LC classification.
Blinding	As life history and locomotory capacity categorisation was conducted before we inferred the branch lengths, blinding was not relevant for our study.

Did the study involve field work? ☐ Yes ☒ No

Reporting for specific materials, systems and methods

We require information from authors about some types of materials, experimental systems and methods used in many studies. Here, indicate whether each material, system or method listed is relevant to your study. If you are not sure if a list item applies to your research, read the appropriate section before selecting a response.

Materials & experimental systems

n/a	Involved in the study	
☒	☐	Antibodies
☒	☐	Eukaryotic cell lines
☒	☐	Palaeontology and archaeology
☒	☐	Animals and other organisms
☒	☐	Clinical data
☒	☐	Dual use research of concern

Methods

n/a	Involved in the study	
☒	☐	ChIP-seq
☒	☐	Flow cytometry
☒	☐	MRI-based neuroimaging
