# Supplementary material for: Mitogenomic evolutionary rates in bilateria are influenced by parasitic lifestyle and locomotory capacity
Source: Nat Commun. 2023 Oct 9;14:6307. doi: 10.1038/s41467-023-42095-8 (PMC10562372; doi:10.1038/s41467-023-42095-8)
Supplement: Supplementary file 4 — Description of Additional Supplementary Files [file 41467_2023_42095_MOESM4_ESM.pdf]

Description of Additional Supplementary Files

Supplementary Data 1
Description: The main dataset (Worksheet 1), exact p-values and other details for pairwise statistical comparisons in Figure 2 (Worksheet 2), details of lmekin and brms analyses (Worksheet 3), correlation values between branch lengths of different phylograms (Worksheet 4).
